# Supplementary material for: Relationships among thriving at work, organisational commitment and job satisfaction among Chinese front-line primary public health workers during COVID-19 pandemic: a structural equation model analysis
Source: BMJ Open. 2022 Jun 7;12(6):e059032. doi: 10.1136/bmjopen-2021-059032 (PMC9174534; doi:10.1136/bmjopen-2021-059032)
Supplement: Author's manuscript [file bmjopen-2021-059032.draft_revisions.pdf]

BMJ Open is committed to open peer review. As part of this commitment we make the peer review history of every article we publish publicly available.

When an article is published we post the peer reviewers' comments and the authors' responses online. We also post the versions of the paper that were used during peer review. These are the versions that the peer review comments apply to.

The versions of the paper that follow are the versions that were submitted during the peer review process. They are not the versions of record or the final published versions. They should not be cited or distributed as the published version of this manuscript.

BMJ Open is an open access journal and the full, final, typeset and author-corrected version of record of the manuscript is available on our site with no access controls, subscription charges or pay-per-view fees (http://bmjopen.bmj.com).

If you have any questions on BMJ Open's open peer review process please email info.bmjopen@bmj.com

BMJ Open

Relationship between thriving at work, organizational commitment and job satisfaction among primary public health workers during pandemic: A structural equation model analysis

Journal:	*BMJ Open*
Manuscript ID	bmjopen-2021-059032
Article Type:	Original research
Date Submitted by the Author:	08-Nov-2021

Complete List of Authors:	Yi, Mo; Shandong University Cheeloo College of Medicine,<br>Jiang, Di; Peking Union Medical College Hospital Eastern Branch<br>Jia, Yuanmin; Shandong University, School of Nursing and Rehabilitation<br>Zhang, Zeyi ; Shandong University Cheeloo College of Medicine, School of Nursing and Rehabilitation<br>Wang, Jingjing ; Shandong University Cheeloo College of Medicine, School of Nursing and Rehabilitation<br>Zhao, Baosheng; Shandong University Cheeloo College of Medicine, School of Nursing and Rehabilitation<br>Guo, Lei; Shandong University Cheeloo College of Medicine, Centre for Health Management and Policy Research, School of Public Health<br>Chen, Ou; Shandong University Cheeloo College of Medicine, School of Nursing and Rehabilitation

Keywords:	COVID-19, Health & safety < HEALTH SERVICES ADMINISTRATION & MANAGEMENT, Human resource management < HEALTH SERVICES ADMINISTRATION & MANAGEMENT, Risk management < HEALTH SERVICES ADMINISTRATION & MANAGEMENT, PUBLIC HEALTH

SCHOLARONE™ Manuscripts

I, the Submitting Author has the right to grant and does grant on behalf of all authors of the Work (as defined in the below author licence), an exclusive licence and/or a non-exclusive licence for contributions from authors who are: i) UK Crown employees; ii) where BMJ has agreed a CC-BY licence shall apply, and/or iii) in accordance with the terms applicable for US Federal Government officers or employees acting as part of their official duties; on a worldwide, perpetual, irrevocable, royalty-free basis to BMJ Publishing Group Ltd ("BMJ") its licensees and where the relevant Journal is co-owned by BMJ to the co-owners of the Journal, to publish the Work in this journal and any other BMJ products and to exploit all rights, as set out in our licence.

The Submitting Author accepts and understands that any supply made under these terms is made by BMJ to the Submitting Author unless you are acting as an employee on behalf of your employer or a postgraduate student of an affiliated institution which is paying any applicable article publishing charge ("APC") for Open Access articles. Where the Submitting Author wishes to make the Work available on an Open Access basis (and intends to pay the relevant APC), the terms of reuse of such Open Access shall be governed by a Creative Commons licence – details of these licences and which Creative Commons licence will apply to this Work are set out in our licence referred to above.

Other than as permitted in any relevant BMJ Author's Self Archiving Policies, I confirm this Work has not been accepted for publication elsewhere, is not being considered for publication elsewhere and does not duplicate material already published. I confirm all authors consent to publication of this Work and authorise the granting of this licence.

For peer review only

Title Page

Title: Relationship between thriving at work, organizational commitment and job satisfaction among primary public health workers during pandemic: A structural equation model analysis

Mo Yi[1], Di Jiang[2], Yuanmin Jia[1], Zeyi Zhang[1], Jingjing Wang[1], Baosheng Zhao[1], Lei Guo[3], **Ou Chen**[1]* **（Corresponding author）**

Mo Yi and Di Jiang are co-first authors of the article

[1] School of Nursing and Rehabilitation, Cheeloo College of Medicine, Shandong University, Jinan, 250012, China.

[2] Peking Union Medical College Hospital, Beijing, 100730, China.

[3] Centre for Health Management and Policy Research, School of Public Health，Cheeloo College of Medicine, Shandong University, Jinan, 250012, China.

***Corresponding author:** Ou Chen, School of Nursing and Rehabilitation, Cheeloo College of Medicine, Shandong University, No.44 Wenhua West Road, Lixia District, Jinan City, Shandong Province, China,

E-mail: chenou@sdu.edu.cn, Tel: (+0086) 15634048920

Keywords: Thriving at work; organizational commitment; job satisfaction; public health workers; structural equation modeling.

For peer review only

Word count: 4030 (excluding title page, references, figures and tables)

Acknowledgments

This study was supported by The Natural Science Foundation of China (82172543); The Natural Science Foundation of Shandong Province (ZR2020MH006); The Key Research and Development Program of Shandong Province (2019GSF108198).

Relationship between thriving at work, organizational commitment and job satisfaction among primary public health workers during pandemic: A structural equation model analysis

Abstract

Purpose: To understand the mediating role and influence mechanism of organizational commitment between thriving at work and job satisfaction among primary public health workers (PHWs) under the normalization of COVID-19 pandemic prevention and control, and to provide a theoretical basis for improving job satisfaction and career stability of primary PHWs.

Methods: We conduct a cross-sectional survey of 601 primary PHWs in 20 primary care units in various part of China using the job satisfaction scale, thriving at work scale, and organizational commitment scale with a random sampling method, and AMOS 26.0 was used to develop a structural equation model.

Results: In the background of severe pandemic prevention and control, the thriving at

For peer review only

work of primary PHWs was 3.17±0.65, and job satisfaction were 3.05±0.69; the scores of their thriving at work, organizational commitment and job satisfaction were all significantly correlated (P<0.01); the results of the structural equation model indicated that organizational commitment had a significant partial mediating effect between thriving at work and job satisfaction, with an overall effect value of 0.867, and the mediated effect value was 0.422, accounting for 48.7% of the total effect size.

Conclusion: Thriving at work and job satisfaction of primary PHWs are at moderate levels, and thriving at work not only affects job satisfaction directly, but also indirectly through organizational commitment. Healthcare policy makers can promote job satisfaction among PHWs by improving their thriving at work and organizational commitment.

Keywords: Thriving at work; organizational commitment; job satisfaction; primary; public health workers; structural equation modeling.

Strengths and limitations of this study

- The current level of job satisfaction among primary PHWs in China during pandemic was moderate and encountered numerous challenges.
- This study can make health-related policy makers aware of the significance of primary PHWs in the fight against COVID-19.
- Organizational commitment mediated significantly between primary PHWs' thriving at work and job satisfaction.
- Targeted measures should be carried out to enhance organizational commitment and form a good working atmosphere in the workplace, and ultimately promote staff's passion and improve their career stability.

1.Introduction

In early 2020, an epidemic called COVID-19 suddenly swept the world, now causing millions of infections and tens of thousands of deaths worldwide. WHO

For peer review only

identified the outbreak as a Public Health Emergency of International Concern (PHEIC), which poses a huge threat to people's health and the development of national economic[1, 2]. China's primary health care units (such as community health service centers, community hospitals, township/village healthcare stations, etc.) are the first line of defense against the COVID-19 epidemic and have played an important role in the battle against the epidemic[3].

Public health is an important part of China's primary healthcare service system and an important force in improving the health status of community residents. Primary PHWs are responsible for the treatment of common and multiple diseases, prevention, healthcare and rehabilitation and other basic medical services[4]. As the gatekeepers of health in epidemic prevention and control, since the outbreak, primary PHWs have not only completed heavy workloads, but also risked being infected while working on the front line[5]. They are in a state of chronic psychological stress and gradually experience emotional exhaustion of their profession, and their personal physical, energetic, and emotional strengths are in a state of overload, which adversely affects both their job satisfaction and thriving at work[6].

Job satisfaction is the degree to which an individual is satisfied with the job itself and its related aspects in the course of working in an organization[7]. Vladica's empirical study, based on previous research, subdivided job satisfaction into internal and external satisfaction[8]. Nowadays, more and more scholars are using job satisfaction as the best predictor of employees' self-fulfillment of their professional values[9]. Unlike the development model of large general hospitals, primary care units in China are faced with multiple challenges such as smaller platform with limited and fewer opportunities, lower monthly salary, shortage in staff resource, and weaker medical service delivery capacity[10]. The current epidemic prevention and control in China is steady but still unstable and unpredictable, and the current status of job satisfaction of primary PHWs with their occupation is one of the focuses of this study.

"Thriving at work" represents positive behaviors in an individual's daily work, such as continuous learning and work dynamics, that ultimately contribute to the upward and flourishing development of the organization[11]. Spreitzer considers thriving

For peer review only

at work to be the optimistic subjective experience that an individual goes through at work, including the experience of learning and the experience of vitality[12]. In other words, these two dimensions reflect the emotional (vitality) and cognitive (learning) nature of the psychological experience of an individual's career. A social embedding model of thriving at work suggests that job satisfaction is a key manifestation of thriving at work[13]. Thriving at work is also an important factor influencing the personal career development and planning of healthcare providers, accordingly contributing to their professional fulfillment and maintaining physical and mental health[14]. Zhao's study pointed out that healthcare providers in China who lacked thriving at work often led to low job satisfaction, which affected their career enthusiasm and stability[15]. It indicates a highly close correlation between job satisfaction and thriving at work, both of which will be directly related to the level of quality of healthcare services.

Organizational commitment refers to the willingness of employees to participate wholeheartedly in the work of the organization as their "unilateral commitment" to the organization increases[16]. Meyer and Allen argue that organizational commitment is a multidimensional concept that consists of three components: affective, continuance, and normative commitment[17]. The level of organizational commitment increases with intrinsic value, which is another expression of thriving at work. A study concentrated on the group of public health nurses noted that organizational commitment was a significant positive predictor of their job satisfaction[18]. In addition, there are studies that confirm that the three dimensions of organizational commitment are also among the important factors that influence individual's job satisfaction[19, 20].

Based on the original research findings, it is known that job satisfaction, thriving at work and organizational commitment are the key factors which would affect person's career stability and professional identity of primary PHWs. However, the inherent influence mechanism among the three variables of PHWs is still unclear. And currently, most existing studies have explored the job satisfaction of physicians or nurses in large hospitals, with less attention paid to the public health personnel group, especially for the grassroot group. Therefore, this study is equipped with some scientific research value for human resource in the field of PHWs.

For peer review only

The first aim of this study was to assess the levels of thriving at work and job satisfaction among primary PHWs during the COVID-19 pandemic; the second aim was to construct structural equation models for these three variables of job satisfaction, thriving at work, and organizational commitment among primary PHWs, empirically analyze their quantitative relationships, as well as give clinical and practical suggestions for further research. This is crucial for improving job satisfaction and stabilizing the workforce of primary PHWs under the normalization of epidemic prevention and control.

2.Methods

2.1 Participants of the study

This study was a cross-sectional study. A random sampling method was used to survey 601 primary PHWs in 20 primary care units (community health service centers and rural health centers) within China during May to October 2020.

Inclusion criteria for participants: ①years of work in primary care units ≥ 1 year; ②having participated in front-line work against the COVID-19 epidemic for more than 1 month; ③their professional qualification is the licensed PHWs. Exclusion criteria were: ①interns and trainees; ②those whose work sites were non-primary care units ③licensed doctor or nurses practitioner with qualification of public health. A total of 650 questionnaires were distributed in this survey, and 601 valid questionnaires were finally returned, with a valid return rate of 92.46%.

2.2 Instruments and Measurement

2.2.1 Socio-demographic information questionnaire

After referring to previous literature and expert consultation, we identified the following demographic information as the study variables: including the gender, age, education background, types of working units (community healthcare service center, township/village health center), years of working and professional titles of the participants.

For peer review only

2.2.2 Job satisfaction questionnaire

The Minnesota Satisfaction Questionnaire (MSQ), a short form scale developed by Weiss, Dawis, England & Lofquist[21], contains two dimensions: internal and external satisfaction. The MSQ has 20 items and is scored on a 5-point Likert scale, with "very dissatisfied", "dissatisfied", "fair", "satisfied", "very satisfied" and "satisfied" respectively." The higher the score, the higher the satisfaction level of primary PHWs with their work. Previous studies have shown that the Cronbach alpha coefficient of this scale is 0.87-0.92[22, 23], and in this study the Cronbach alpha coefficient was 0.90.

2.2.3 Organizational commitment questionnaire

The Organizational commitment questionnaire (QCQ), developed by Allen and Meyer[24] was used in this study to measure the organizational commitment of primary PHWs. It contains three dimensions: affective commitment, normative commitment, and sustaining commitment, and each dimension includes six items. All items are measured on a Likert 5-point scale from 1 (strongly disagree) to 5 (strongly agree). The Cronbach alpha for each dimension of the scale was 0.87, 0.75, and 0.79. The total scale score was the sum of the items and ranged from 18 to 90, with higher scores representing higher levels of employee's organizational commitment[25]. In this study, the Cronbach alpha coefficient of the scale was 0.917.

2.2.4 Thriving at work scale

The Thriving at work scale developed by Porath[26] was used, which has 10 items and measures two dimensions of thriving at work, vitality and learning, each containing 5 items. This is a Likert 5 scale of "1 to 5" represents "very unlikely ~ very likely", respectively. The higher the score, the higher the sense of thriving at work of the participants. The Chinese version of the questionnaire has been shown to have good reliability and validity in the Chinese teacher population[27]. In this study, the overall internal consistency coefficient α of the scale was 0.92, and the Cronbach′s α coefficients for the vitality and learning dimensions were 0.87 and 0.89, respectively.

For peer review only

2.3 Data collection

This study was conducted in an independent and anonymous self-administered manner on site. The investigators consisted of a cross-disciplinary team of graduate students with different professional backgrounds from the disciplines of public health, nursing, and psychology, respectively. Uniform training and rigorous assessment of the investigators were conducted before the survey began, and the study participants all signed an informed consent form priority to the study. The questionnaires were distributed together by the members in cooperation with the leaders of the primary healthcare units, and they were explained using a uniform guideline. The questionnaires were carefully checked by the investigators after the participants had completed them and were collected in time. The study was approved by the Ethics Committee of University.

2.4 Patient and Public Involvement

No patient involved

2.5 Statistical analysis

Epi data 3.1 was used to establish the database and SPSS 21.0 was used to analyze the data descriptively. t-test and one-way ANOVA were used to compare the differences between variables on the socio-demographic characteristics of the study population. Pearson correlation analysis was used to clarify the correlation between organizational commitment, job satisfaction and thriving at work among primary PHWs, with a p-value <0.05 being considered a statistically significant difference. Based on the correlation analysis, AMOS 26.0 was used to develop a structural equation model to analyze the inherent relationship between the three variables. The path coefficients between the latent variables were tested using path analysis, and the parameter estimation was performed using the great likelihood method, assuming that the significance of the mediating effect of organizational commitment in the model was calculated using Bootstrap analysis with 2000 samples with the test level set at $\alpha=0.05$,

For peer review only

and if the 95% confidence interval of the standardized path coefficients did not contain 0, then the mediating effect was significant. The structural equation model can be considered well-fitted when all path coefficients satisfy the criteria of Hu and Bentler's study[28].

3. Results

3.1 General demographic

A total of 601 primary PHWs who had participated in the frontline of epidemic fighting were surveyed. The average age of the participants was (36. 61±8.95) years old, with the majority being female (82.9%). The education background of all participants was mainly college or above (94.0%), the average working years were (14. 31±6.21) years, and their professional titles are mainly junior level in healthcare (72.3%).

3.2 Analysis of common method bias test

In this study, common method bias test was conducted using Harman's one-way test and exploratory factor analysis was performed without rotation. The results showed that there were 14 factors with characteristic roots greater than 1. The variance rate explained by the first factor of job satisfaction was 36.73%, which was less than the critical value of 40%, indicating that there was no serious problem of common method bias in this study[29].

3.3 Differences in thriving at work and job satisfaction scores of primary public health workers on demographic variables

The results showed that for primary PHWs, there were statistical differences in thriving at work on three demographic variables: gender, education, and years of working. Female primary PHWs had higher thriving at work than male, additionally, the lowest level of thriving at work was observed in the group of the year of working more than 20 years; and the group of lower professional titles showed more thriving.

For peer review only

There were statistical differences in job satisfaction among primary PHWs on three demographic variables: gender, education. Female PHWs had higher satisfaction than males in the job; PHWs with higher years of working had higher level of job satisfaction; PHWs with higher education had lower job satisfaction, as detailed in **Table 1**.

Table 1 Demographic differences in Thriving at Work and Job Satisfaction among primary PHWs

Variables	Scores of T W		Scores of JS	
	scores	*t/F* value	scores	*t/F* value
**Gender**		-0.813**		-1.351**
Male	3.13±0.70		3.91±0.80	
Female	3.18±0.64		3.07±0.67	
**Age**		0.126		0.066
<30 years old	3.16±0.74		3.04±0.74	
30-45 years old	3.19±0.59		3.06±0.67	
>45 years old	3.16±0.63		3.05±0.68	
**Years of work**		0.267*		0.021*
<10 years	3.15±0.69		3.05±0.72	
10 -20 years	3.20±0.61		3.03±0.68	
>20 years	3.17±0.67		3.06±0.66	
**Educational background**		0.396*		0.785**
College and below	3.16±0.54		3.08±0.57	
University Undergraduate	3.12±0.65		3.02±0.68	
Postgraduate and above	3.05±0.61		3.96±0.46	
**Professional title**		0.311		0.655
Junior	3.05±0.72		3.05±0.72	
Intermediate	3.03±0.68		3.03±0.68	
Advanced	3.04±0.66		3.06±0.66	

Abbreviations: TW, thriving at work; OC, organizational commitment; JS, job satisfaction.

For peer review only

Note: *$P<0.05$, **$P<0.01$.

3.4 Correlation between organizational commitment, job satisfaction and thriving at work among primary PHWs

The results showed that the mean score of thriving at work score of the 601 surveyed primary PHWs was (3.17±0.65). The mean score of their organizational commitment score was (2.93±0.73). and the mean score of their job satisfaction score was (3.05±0.69). Pearson's correlation analysis revealed that the thriving at work of primary PHWs was positively correlated with organizational commitment (r=0.715, P<0.001) and highly positively correlated with job satisfaction (r=0.804, P<0.001); furthermore, there was a high positive correlation between organizational commitment and job satisfaction (r=0.848, P<0.001), see **Table 2**.

Table 2 Correlation between thriving at work, organizational commitment and job satisfaction among primary public health workers

Name of variables	Mean ± SD	TW	OC	JS
TW	3.17±0.65	1	—	—
OC	2.93±0.73	0.715**	1	—
JS	3.05±0.69	0.804**	0.848**	1

Abbreviations: TW, thriving at work, OC, organizational commitment, JS, job satisfaction
Note: *$P<0.05$, **$P<0.01$.

3.5 Structural equation modeling of organizational commitment, job satisfaction, and thriving at work among primary PHWs

In order to investigate the influence mechanism among the variables in depth, after controlling for the gender, age, education background, and years of work of the study participants. A structural equation model was constructed with thriving at work as the predictor variable, organizational commitment as the mediating variable, and job satisfaction as the outcome variable. AMOS 26.0 software was used to conduct the validation analysis of the hypothesized model, and the results showed that each path

For peer review only

coefficient was statistically significant (p<0.001), as shown in **Figure 1**. The model fit statistics were: chi-squared degrees of freedom (χ^2/df) =3.416, GFI=0.982, AGFI=0.953, NFI=0.993, CFI=0.995, IFI=0.995, and RMSEA=0.063 (95% confidence interval 0.042,0.086), respectively. All the above fit indices are within the acceptable range and the model fits well, as shown in **Table 3**.

Table 3 Index Evaluation System and Fitting Results of Structural Equation Model Adaptability

Fit Index	χ^2/df	GFI	AGFI	RMSEA	IFI	CFI	NFI
Theoretical Model	<3	>0.9	>0.9	<0.08	>0.9	>0.9	>0.9
Regression criterion	3.416	0.982	0.953	0.063	0.995	0.995	0.993

Abbreviations: df, degree of freedom; GFI, goodness-of-fit index; AGFI, adjusted goodness-of-fit index; RMSEA, root-mean-square error of approximation; IFI: Incremental Fit Index; CFI, comparative fit index; NFI, normed fit index.

Bootstrap method was used to test the significance of the mediating role of organizational commitment between thriving at work and job satisfaction. Repeated samples were taken 2000 times from the original data and 95% confidence intervals were calculated. The results showed that the standardized effect value of the mediating effect of organizational commitment between thriving at work and job satisfaction was 0.422 with 95% *CI* (0.276,0.561); the standardized effect value of the total effect was 0.867 with 95% *CI* (0.781,0.920), both of which did not contain 0, indicating that the differences in all effect paths were statistically significant. The calculated mediated effect size was 48.7% of the total effect size, and the specific path coefficients of the model are shown in **Table 4**.

Table 4 Pathway coefficients of structural equation model (n=601)

For peer review only

Pathways	Std. estimate	95% CI	SE	P-value
**Total effect**				
	0.867	(0.781,0.920)		0.002**
**Direct effect**				
TW→JS	0.445	(0.269,0.640)	0.098	0.001**
TW→OC	0.765	(0.662,0.844)	0.046	<0.01**
OC→JS	0.552	(0.364,0.726)	0.096	0.001**
**Indirect effect**				
TW→OC→JS	0.422	(0.276,0.561)	0.070	0.001**

Abbreviations: TW, thriving at work; OC, organizational commitment; JS, job satisfaction; Std. Estimate, Standardized estimate; CI, Confidence interval; SE, Standard error.

Note: **P*< 0.05, ***P*< 0.01.

4. Discussion

This study found that during the COVID-19 pandemic, primary PHWs in China had a moderate level of job satisfaction. Adequate thriving at work can directly enhance job satisfaction. At the same time, thriving at work can also indirectly influence job satisfaction through organizational commitment.

The current status of thriving at work and job satisfaction among primary PHWs

Job satisfaction is an important indicator of the quality of work done by primary PHWs, which has an important impact on their physical and mental health development[30]. However, since the outbreak of the COVID-19 epidemic, a few research has been reported on the job satisfaction of the primary PHWs workforce in China. In the context of the unstable control of the COVID-19 epidemic in domestic China, when small-scale outbreaks occur in localized areas, primary PHWs are on the front line of the fight against the epidemic and are required to respond quickly to their areas of responsibility, such as conducting epidemiological investigations of infected persons, running residential nucleic acid testing and thorough disinfection of the environment[31].

For peer review only

As a result, they are exposed to greater work intensity, physical and mental stress caused by work-family conflicts and so on, which seriously affects the level of job satisfaction of primary PHWs[32].

The results of this study showed that the thriving at work score of primary PHWs was 3.17±0.65. Besides, the score of job satisfaction was 3.05±0.69, which was lower than the score levels of PHWs in Nigeria and Iran[33, 34]. The results of the ANOVA analysis showed that there were statistically significant differences in the job satisfaction scores of primary PHWs in variables of gender, years of working, professional title and education background. That is, primary PHWs with higher education, fewer years of work experience and lower job titles are at a relatively lower level of job satisfaction. On the one hand, this may be due to the fact that grassroots jobs offer fewer development opportunities, lower incomes and inadequate supporting mechanisms, resulting in grassroots PHWs with higher education not being able to give full play to their knowledge, thus limiting their long-term career development and resulting in a situation of lower job satisfaction for them[35]. On the other hand, because there is a close correlation between years of service and job title as an indicator, when employees have lower years of service, their titles are correspondingly lower, resulting in very limited income levels and social status, leading to a lower level of job satisfaction for this group as well[36].

Correlation and structural equation modeling between organizational commitment, thriving at work, and job satisfaction among primary PHWs

Many previous studies have shown a positive relationship between healthcare providers' organizational commitment and their job satisfaction, while the relationship between thriving at work and job satisfaction has not been confirmed in a primary PHWs population. The results of Pearson's analysis showed that thriving at work, organizational commitment, and job satisfaction, these three variables were significantly positively correlated (p<0.01), which could provide new ideas for future research. Firstly, the integrated model of personal growth for “Thriving at work”[37] suggests that a significant output of thriving at work is the indicator of job satisfaction.

For peer review only

A meta-analysis that included 21,739 employees has found that teachers with high levels of thriving at work would exhibit higher levels of job satisfaction[38]. In addition, thriving at work is one of the decisive factors for the sustainability of public health personnel and their health-related organizations. Individual's thriving at work focuses on two domains, vitality and learning, in daily work[39]. Primary PHWs can create a positive work ambiance through active learning in a variety of ways, which can be very beneficial for both the department and the health care unit. In addition, vitality is an important factor in maintaining thriving at work and can help individuals adapt positively to difficulties in their situation, especially during the COVID-19 pandemic when the situation was volatile, risky, and challenging[40]. Energetic primary PHWs are less susceptible to negative emotions and feel more social support from all sides, would buffer their severe job burnout and improve their job satisfaction during the pandemic[41].

Secondly, the Pearson results also indicated that organizational commitment of PHWs showed a high positive relationship with their job satisfaction. Organizational commitment refers to the degree of employees' psychological attachment to the organization[42]. This study shows that PHWs with high organizational commitment have a deep attachment to their healthcare units and a strong sense of responsibility and mission for their jobs. According to Meyer, organizational commitment can be subdivided into three types: affective commitment, continuance commitment, and normative commitment[43]. The correlation between these three types and job satisfaction can be reflected as follows: the higher the affective commitment, the higher the identification with the organization's goals, values and culture, and the higher the satisfaction with the job. The higher the normative commitment, the higher the responsibility to the organization, the higher the psychological satisfaction that active work can bring to the individual, and the higher their job satisfaction[44, 45]. Health-related policy authority should pay attention to the primary PHWs' identification with organizational values and culture, education on social norms and professional ethics as a code of conduct, helping them to develop practical career plans, and attaching importance to their professional growth, which is crucial to the development of organizational commitment[46].

For peer review only

This study also found that the relationship between thriving at work and job satisfaction was also influenced by organizational commitment to some extent. The results of the structural equation model showed that organizational commitment partially mediated the relationship between thriving at work and job satisfaction, with a mediating effect size of -0.35, accounting for 57.38% of the total effect, indicating that organizational commitment is an important factor in the relationship between thriving at work and job satisfaction among primary PHWs, and that thriving at work can also serve as an important predictor of job satisfaction.

Further analyzing the intrinsic mechanism, according to the social embedding model of thriving at work proposed by Spreitzer[47], the work context in which an individual is embedded largely determines individual's level of work thriving. From the outbreak of the epidemic in early 2020 to the present, the current situation of fighting the COVID-19 is still uncertain and a series of serious challenges remain. Such working environment will inevitably have a negative impact on the thriving of primary PHWs, leading to their susceptibility to psychological stress situations such as fear, anxiety and neurosis, which in turn reduces their job satisfaction and will be detrimental to the development of their ability to respond to public health emergent events[5]. It is a scientific approach that healthcare managers should enhance the level of organizational commitment of PHWs by stimulating their innovative motivation and behavior and strengthening their organizational culture. In addition, they can be encouraged to regain their self-worth and professional fulfillment in the fight against the epidemic, which will ultimately lead to the improvement of job quality and satisfaction.

In conclusion, grassroots PHWs are on the front line of the battle against the COVID-19 pandemic, and they have made the greatest sacrifice for this global public health emergency[32]. This study explored the intrinsic link between thriving at work and job satisfaction by constructing a structural equation model with organizational commitment as a mediating variable. thriving can promote career sustainability among primary PHWs and can have a direct impact on their job satisfaction, as well as an indirect impact on their job satisfaction by increasing the level of organizational commitment. This study provides a scientific and theoretical basis for better stabilizing

For peer review only

the talent pool of primary PHWs in China, improving the service quality of primary public health and medical care in China, and optimizing the allocation of primary medical resources.

Limitation

Firstly, some participants in this study may have had recall bias when completing the scales. Secondly, this study was a multicenter study conducted in China for primary healthcare facilities. Therefore, caution should be exercised when generalizing the results of this study to other countries, especially to developed countries. Finally, because this study was a cross-sectional study, there was no relevant causality. Future longitudinal studies with larger samples and incorporating more variables are needed to validate the results of this study.

5. Conclusion

This study found that thriving at work has a direct contribution to job satisfaction of primary PHWs through structural equation modeling, while organizational commitment as a mediating variable can improve job satisfaction. In the context of the COVID-19 pandemic, healthcare policy makers should pay high attention to primary PHWs and should adopt incentives to increase their thriving at work and organizational commitment so as to effectively activate their job satisfaction and sense of accomplishment, which are essential to maintain the career stability of primary PHWs.

Author Contributions

Conceived and designed the experiments: Mo Yi, Di Jiang, Ou Chen.

Performed the experiments: Yuanmin Jia, Jingjing Wang, Lei Guo, Zeyi Zhang.

Analyzed the data: Mo Yi, Yuanmin Jia, Mo Yi, Di Jiang

Contributed reagents/materials/analysis tools: Yuanmin Jia, Baosheng Zhao.

Wrote the manuscript: Mo Yi.

Mo Yi and Di Jiang are co-first authors of the article

For peer review only

Acknowledgments

We are particularly grateful to the coordinators who assisted us, especially all the primary PHWs who participated in this study and supported the study during the busy period of epidemic prevention and control.

Funding

This study was supported by The Natural Science Foundation of China (82172543); The Natural Science Foundation of Shandong Province (ZR2020MH006); The Key Research and Development Program of Shandong Province (2019GSF108198).

Disclosure

The authors report no conflicts of interest in this work.

Data availability statement

The data or models generated or used during the study are available from the corresponding author by request.

Ethics statement

The study was approved by the ethic board of school of nursing and rehabilitation, Shandong university, China. All participants provided written informed consent.

References:

1. Lancet T. COVID-19: too little, too late? *Lancet (London, England).* 2020(No.10226):755.

2. Liu C, Zhao Y, Okwan-Duodu D, Basho R, Cui X. COVID-19 in cancer patients: risk, clinical features, and management. *Cancer Biol Med.* 2020(No.3):519-527.

For peer review only

3. Wang H, Tang C, Zhao S, Meng Q, Liu X. Job Satisfaction among Health-Care Staff in Township Health Centers in Rural China: Results from a Latent Class Analysis. *Int J Environ Res Public Health.* 2017;14(10).

4. Shi LSL, Song KSK, Rane SRS, Sun XSX, Li HLH, Meng QMQ. Factors associated with job satisfaction by Chinese primary care providers. *Prim Health Care Res.* 2014(No.1):46-57.

5. Li J, Xu J, Zhou H, et al. Working conditions and health status of 6,317 front line public health workers across five provinces in China during the COVID-19 epidemic: a cross-sectional study. *Bmc Public Health.* 2021;21(1):106.

6. Jing Zhang YWJX. Prevalence of mental health problems and associated factors among front-line public health workers during the COVID-19 pandemic in China: an effort-reward imbalance model-informed study. *BMC psychology.* 2021(No.1):55.

7. Kuo HT, Lin KC, Li IC. The mediating effects of job satisfaction on turnover intention for long-term care nurses in Taiwan. *J Nurs Manag.* 2014;22(2):225-233.

8. Veličković VMA, Višnjić AAB, Jović SA, et al. Organizational commitment and job satisfaction among nurses in Serbia: A factor analysis(Article). *Nurs Outlook.* 2014(No.6):415-427.

9. Eacute P, Maximilian, Haefner N, Golden T. Clarifying CSE on Job Performance: The Roles of Innovative Work Behavior & Transformational Leaders. *Academy of Management Annual Meeting Proceedings.* 2018(No.1):1.

10. Wang LWL, Wang ZWZ, Ma QMQ, Fang GFG, Yang JYJ. The development and reform of public health in China from 1949 to 2019. *Globalization & Health.* 2019(No.1):45.

11. Abid GAG, Zahra IZI, Ahmed AAA. Promoting thriving at work and waning turnover intention: A relational perspective. *Future Business Journal.* 2016(No.2):127-137.

12. Spreitzer GCFP, Sutcliffe KCFP, Dutton JCFP, Sonenshein SCFP, Grant AMCF. A Socially Embedded Model of Thriving at Work. *Organ Sci.* 2005(NO.5):537-549.

13. Abid G, Khan B, Hong MC. Thriving at Work: How Fairness Perception Matters for Employee's Thriving and Job Satisfaction. *Academy of Management Annual Meeting Proceedings.* 2016(No.1):1.

14. Abid GGAH, Zahra IIZY, Ahmed ADNE. Mediated Mechanism of Thriving at Work between Perceived Organization Support, Innovative Work Behavior and Turnover Intention. *Pakistan Journal of Commerce & Social Sciences.* 2015(No.3):982-998.

15. Zhao SH, Shi Y, Sun ZN, et al. Impact of workplace violence against nurses' thriving at work, job satisfaction and turnover intention: A cross-sectional study. *J Clin Nurs.* 2018;27(13-14):2620-2632.

16. Güleryüz GHUD, Güney SHUD, Aydın EMHU, Aşan ÖHUD. The mediating effect of job satisfaction between emotional intelligence and organisational commitment of nurses: A questionnaire survey. *Int J Nurs Stud.* 2008(No.11):1625-1635.

17. Meyer JP, Allen NJ. A three-component conceptualization of organizational commitment. *Hum Resour Manage R.* 1991(NO.1):61-89.

18. Aharon AA, Madjar B, Kagan I. Organizational commitment and quality of life at work among public health nurses in Israel. *Public Health Nurs.* 2019(No.4):534-540.

19. Culibrk JCJ, Delic MDM, Mitrovic SMS, Culibrk DCD. Job Satisfaction, Organizational Commitment and Job Involvement: The Mediating Role of Job Involvement. *Front Psychol.* 2018:132.

20. Eric G Lambert RTSE. The Association of Organizational Justice with Job Satisfaction and Organizational Commitment among Nigerian Correctional Staff. *Int J Offender Ther.* 2021(No.2-3):180-204.

21. Lakatamitou I, Lambrinou E, Kyriakou M, Paikousis L, Middleton N. The Greek versions of the

For peer review only

TeamSTEPPS teamwork perceptions questionnaire and Minnesota satisfaction questionnaire "short form". *Bmc Health Serv Res.* 2020;20(1):587.
22. Li N, Zhang L, Xiao G, Chen J, Lu Q. The relationship between workplace violence, job satisfaction and turnover intention in emergency nurses. *Int Emerg Nurs.* 2019:50-55.
23. Zhang X, Bian L, Bai X, et al. The influence of job satisfaction, resilience and work engagement on turnover intention among village doctors in China: a cross-sectional study. *Bmc Health Serv Res.* 2020;20(1):283.
24. Allen NJUW, Meyer JP. Affective, continuance, and normative commitment to the organization: An examination of construct validity. *J Vocat Behav.* 1996(No.3):252-276.
25. Lin WQA, Yuan LXB, Kuang SYB, et al. Work engagement as a mediator between organizational commitment and job satisfaction among community health-care workers in China: a cross-sectional study. *Psychology, Health and Medicine.* 2020(No.6):666-674.
26. Porath CA, Spreitzer GASU, Gibson CA, Garnett FGA. Thriving at work: Toward its measurement, construct validation, and theoretical refinement. *J Organ Behav.* 2012(No.2):250-275.
27. Yi Yang XL. The Impact of Challenge and Hindrance Stressors on Thriving at Work Double Mediation Based on Affect and Motivation. *Front Psychol.* 2021:613871.
28. Hu L, Bentler PM. Fit indices in covariance structure modeling: Sensitivity to underparameterized model misspecification. *Psychol Methods.* 1998(No.4):424-453.
29. Doty DHSU, Glick WH. Common Methods Bias: Does Common Methods Variance Really Bias Results? *Organ Res Methods.* 1998(NO.4):374-406.
30. Warr P, Inceoglu I. Job Engagement, Job Satisfaction, and Contrasting Associations With Person-Job Fit.[Article]. *J Occup Health Psych.* 2012(No.2):129-138.
31. Huang J, Liu F, Teng Z, et al. Care for the Psychological Status of Frontline Medical Staff Fighting Against Coronavirus Disease 2019 (COVID-19). *Clin Infect Dis.* 2020;71(12):3268-3269.
32. She R, Wang X, Zhang Z, et al. Mental Health Help-Seeking and Associated Factors Among Public Health Workers During the COVID-19 Outbreak in China. *Front Public Health.* 2021;9:622677.
33. Adeniji AA, Osibanjo AO, Iruonagbe TC, Olawande T, Ibidunni AS, Olokundun MA. From job satisfaction to organizational commitment: The mediating influence of perceived treatment of diversity among nigeria’ s public healthcare employees(Article). *Open Access Macedonian Journal of Medical Sciences.* 2019(No.12):2031-2035.
34. Sarabi RE, Javanmard R, Shahrbabaki PM. Study of burnout syndrome, job satisfaction and related factors among health care workers in rural areas of Southeastern Iran. *AIMS Public Health.* 2020(No.1):158-168.
35. Li L, Hu H, Zhou H, et al. Work stress, work motivation and their effects on job satisfaction in community health workers: a cross-sectional survey in China. *Bmj Open.* 2014;4(6):e4897.
36. Lu YLY, Hu XHX, Huang XHX, et al. Job satisfaction and associated factors among healthcare staff: a cross-sectional study in Guangdong Province, China. *Bmj Open.* 2016(No.7):e11388.
37. Spreitzer GM, Porath CL. Self-Determination as a Nutriment for Thriving: Building an Integrative Model of Human Growth at Work. *The Oxford Handbook of Work Engagement, Motivation, and Self-Determination Theory.* 2014.
38. Kleine AKAA, Rudolph CWA, Zacher HAHZ. Thriving at work: A meta‐analysis. *J Organ Behav.* 2019(No.9-10):973-999.
39. Danping Liu SZYW. The Antecedents of Thriving at Work: A Meta-Analytic Review. *Front Psychol.* 2021:659072.

For peer review only

40. Ding YDY, Qu JQJ, Yu XYX, Wang SWS. The Mediating Effects of Burnout on the Relationship between Anxiety Symptoms and Occupational Stress among Community Healthcare Workers in China: A Cross-Sectional Study. *Plos One.* 2014(No.9):e107130.

41. Stone KW, Kintziger KW, Jagger MA, Horney JA. Public Health Workforce Burnout in the COVID-19 Response in the U.S. *Int J Environ Res Public Health.* 2021;18(8).

42. Oyinlade AO, Christo ZJ. Path Analysis between Organizational Size and Forms of Organizational Commitment. *American Journal of Industrial and Business Management.* 2020(第10期).

43. Meyer JP, Allen NJ. A three-component conceptualization of organizational commitment. *Hum Resour Manage R.* 1991(NO.1):61-89.

44. Asiri SA, Rohrer WW, Al-Surimi K, Da'Ar OO, Ahmed A. The association of leadership styles and empowerment with nurses' organizational commitment in an acute health care setting: a cross-sectional study. *BMC Nurs.* 2016;15:38.

45. Solinger ON, van Olffen W, Roe RA. Beyond the three-component model of organizational commitment. *J Appl Psychol.* 2008;93(1):70-83.

46. Du Z, You H, Zhou H, et al. Difficulties encountered by public health workers in COVID-19 outbreak: a cross-sectional study based on five provinces. *Bmc Health Serv Res.* 2021(No.1):656.

47. Sreitzer GA, Porath CLB, Gibson CBC. Toward human sustainability. How to enable more thriving at work.(Article). *Organ Dyn.* 2012(No.2):155-162.

Abbreviations: CC, continuance commitment; NC, normative commitment; AC, affective commitment; IS, internal satisfaction; ES, external satisfaction.

Figure 1 Structural equation model of primary PHWs' thriving at work, organizational commitment, job satisfaction

For peer review only

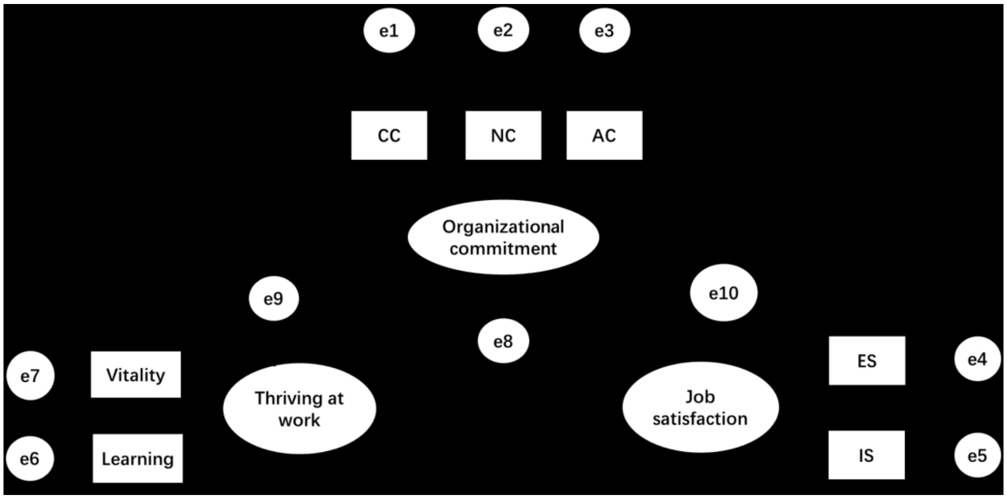

Structural equation model of primary PHWs' thriving at work, organizational commitment, job satisfaction

854x419mm (59 x 59 DPI)

STROBE Statement—Checklist of items that should be included in reports of ***cross-sectional studies***

	Item No	Recommendation	Page No
**Title and abstract**	1	(*a*) Indicate the study's design with a commonly used term in the title or the abstract	2
		(*b*) Provide in the abstract an informative and balanced summary of what was done and what was found	2
**Introduction**			
Background/rationale	2	Explain the scientific background and rationale for the investigation being reported	3
Objectives	3	State specific objectives, including any prespecified hypotheses	5
**Methods**			
Study design	4	Present key elements of study design early in the paper	5
Setting	5	Describe the setting, locations, and relevant dates, including periods of recruitment, exposure, follow-up, and data collection	5
Participants	6	(*a*) Give the eligibility criteria, and the sources and methods of selection of participants	5
Variables	7	Clearly define all outcomes, exposures, predictors, potential confounders, and effect modifiers. Give diagnostic criteria, if applicable	5,6
Data sources/ measurement	8*	For each variable of interest, give sources of data and details of methods of assessment (measurement). Describe comparability of assessment methods if there is more than one group	7
Bias	9	Describe any efforts to address potential sources of bias	7
Study size	10	Explain how the study size was arrived at	7
Quantitative variables	11	Explain how quantitative variables were handled in the analyses. If applicable, describe which groupings were chosen and why	7
Statistical methods	12	(*a*) Describe all statistical methods, including those used to control for confounding	8
		(*b*) Describe any methods used to examine subgroups and interactions	8
		(*c*) Explain how missing data were addressed	8
		(*d*) If applicable, describe analytical methods taking account of sampling strategy	8
		(*e*) Describe any sensitivity analyses	8
**Results**			
Participants	13*	(a) Report numbers of individuals at each stage of study—eg numbers potentially eligible, examined for eligibility, confirmed eligible, included in the study, completing follow-up, and analysed	9
		(b) Give reasons for non-participation at each stage	9
		(c) Consider use of a flow diagram	
Descriptive data	14*	(a) Give characteristics of study participants (eg demographic, clinical, social) and information on exposures and potential confounders	9
		(b) Indicate number of participants with missing data for each variable of interest	9
Outcome data	15*	Report numbers of outcome events or summary measures	10
Main results	16	(*a*) Give unadjusted estimates and, if applicable, confounder-adjusted estimates and their precision (eg, 95% confidence interval). Make clear which confounders were adjusted for and why they were included	10

For peer review only

For peer review only - http://bmjopen.bmj.com/site/about/guidelines.xhtml

		(*b*) Report category boundaries when continuous variables were categorized	11
		(*c*) If relevant, consider translating estimates of relative risk into absolute risk for a meaningful time period	11
Other analyses	17	Report other analyses done—eg analyses of subgroups and interactions, and sensitivity analyses	12,13
**Discussion**			
Key results	18	Summarise key results with reference to study objectives	13-17
Limitations	19	Discuss limitations of the study, taking into account sources of potential bias or imprecision. Discuss both direction and magnitude of any potential bias	17
Interpretation	20	Give a cautious overall interpretation of results considering objectives, limitations, multiplicity of analyses, results from similar studies, and other relevant evidence	17
Generalisability	21	Discuss the generalisability (external validity) of the study results	17
**Other information**			
Funding	22	Give the source of funding and the role of the funders for the present study and, if applicable, for the original study on which the present article is based	18

*Give information separately for exposed and unexposed groups.

Note: An Explanation and Elaboration article discusses each checklist item and gives methodological background and published examples of transparent reporting. The STROBE checklist is best used in conjunction with this article (freely available on the Web sites of PLoS Medicine at http://www.plosmedicine.org/, Annals of Internal Medicine at http://www.annals.org/, and Epidemiology at http://www.epidem.com/). Information on the STROBE Initiative is available at www.strobe-statement.org.

For peer review only

BMJ Open

Relationship between thriving at work, organizational commitment and job satisfaction among Chinese primary public health workers during pandemic: a structural equation model analysis

Journal:	*BMJ Open*
Manuscript ID	bmjopen-2021-059032.R1
Article Type:	Original research
Date Submitted by the Author:	07-Mar-2022
Complete List of Authors:	Yi, Mo; Shandong University, Cheeloo College of Medicine, School of Nursing and Rehabilitation<br>Jiang, Di; Peking Union Medical College Hospital Eastern Branch, Department of Nursing<br>Jia, Yuanmin; Shandong University, School of Nursing and Rehabilitation<br>Wang, Jingjing ; Shandong University, Cheeloo College of Medicine, School of Nursing and Rehabilitation<br>Zhang, Zeyi ; Shandong University, Cheeloo College of Medicine, School of Nursing and Rehabilitation<br>Zhao, Baosheng; Shandong University, Cheeloo College of Medicine, School of Nursing and Rehabilitation<br>Guo, Lei; Shandong University, Cheeloo College of Medicine, Centre for Health Management and Policy Research; Shandong University, Cheeloo College of Medicine, School of Public Health<br>Chen, Ou ; Shandong University, Cheeloo College of Medicine, School of Nursing and Rehabilitation
**Primary Subject Heading**:	Public health
Secondary Subject Heading:	Health services research, Medical management, Occupational and environmental medicine
Keywords:	COVID-19, Health & safety < HEALTH SERVICES ADMINISTRATION & MANAGEMENT, Human resource management < HEALTH SERVICES ADMINISTRATION & MANAGEMENT, PUBLIC HEALTH

SCHOLARONE™
Manuscripts

I, the Submitting Author has the right to grant and does grant on behalf of all authors of the Work (as defined in the below author licence), an exclusive licence and/or a non-exclusive licence for contributions from authors who are: i) UK Crown employees; ii) where BMJ has agreed a CC-BY licence shall apply, and/or iii) in accordance with the terms applicable for US Federal Government officers or employees acting as part of their official duties; on a worldwide, perpetual, irrevocable, royalty-free basis to BMJ Publishing Group Ltd ("BMJ") its licensees and where the relevant Journal is co-owned by BMJ to the co-owners of the Journal, to publish the Work in this journal and any other BMJ products and to exploit all rights, as set out in our licence.

The Submitting Author accepts and understands that any supply made under these terms is made by BMJ to the Submitting Author unless you are acting as an employee on behalf of your employer or a postgraduate student of an affiliated institution which is paying any applicable article publishing charge ("APC") for Open Access articles. Where the Submitting Author wishes to make the Work available on an Open Access basis (and intends to pay the relevant APC), the terms of reuse of such Open Access shall be governed by a Creative Commons licence – details of these licences and which Creative Commons licence will apply to this Work are set out in our licence referred to above.

Other than as permitted in any relevant BMJ Author's Self Archiving Policies, I confirm this Work has not been accepted for publication elsewhere, is not being considered for publication elsewhere and does not duplicate material already published. I confirm all authors consent to publication of this Work and authorise the granting of this licence.

For peer review only

Title Page

Title: Relationship between thriving at work, organizational commitment and job satisfaction among Chinese primary public health workers during pandemic: a structural equation model analysis

Mo Yi[1], Di Jiang[2], Yuanmin Jia[1], Jingjing Wang[1], Zeyi Zhang[1], Baosheng Zhao[1], Lei Guo[3,4], Ou Chen[1]*

Mo Yi and Di Jiang are co-first authors of the article

[1] School of Nursing and Rehabilitation, Cheeloo College of Medicine, Shandong University, Jinan, 250012, China.

[2] Peking Union Medical College Hospital, Beijing, 100730, China.

[3] Centre for Health Management and Policy Research, Shandong University, Jinan, 250012, China.

[4]School of Public Health, Cheeloo College of Medicine, Shandong University, Jinan, 250012, China

***Corresponding author:** Ou Chen, School of Nursing and Rehabilitation, Cheeloo College of Medicine, Shandong University, No.44 Wenhua West Road, Lixia District, Jinan City, Shandong Province, 250012, China.

E-mail: chenou@sdu.edu.cn, Tel: (+0086) 053188382000

Key words: Public health, Thriving at work, Organizational commitment, Job satisfaction, Structural equation model

Word count

4215(excluding title page, references, figures and tables)

For peer review only

Acknowledgments

This study was supported by The Natural Science Foundation of China (82172543); The Natural Science Foundation of Shandong Province (ZR2020MH006); The Key Research and Development Program of Shandong Province (2019GSF108198).

For peer review only

Relationship between thriving at work, organizational commitment and job satisfaction among Chinese primary public health workers during pandemic: a structural equation model analysis

Abstract

Objectives: This study aims to explore the mediating effect and influence mechanism of organizational commitment on the association between thriving at work and job satisfaction among primary public health workers (PHWs) in China during COVID-19 pandemic.

Design: This study is a cross-sectional written survey.

Setting: We included 20 primary care units across northern province of China.

Participants: 601 PHWs who worked in the primary organizations and against the COVID-19 in the frontline were included.

Methods: We collected the data from the participants' written questionnaire (Minnesota Satisfaction Questionnaire, thriving at work scale, and organizational commitment scale), and programmed AMOS 26.0 to develop a structural equation model (SEM) based on the relationship of three variables.

Results: The scores of thriving at work of primary PHWs was (M=3.17, SD=0.65), and job satisfaction were (M=3.05, SD=0.69); the scores of their thriving at work,

organizational commitment and job satisfaction were all significantly correlated (P<0.01); the SEM indicated that organizational commitment had a significant partial mediating effect between thriving at work and job satisfaction. The overall effect value was 0.867, and the mediated effect value was 0.422, accounting for 48.7% of the total effect size.

Conclusion: Thriving at work and job satisfaction of primary PHWs in China are at moderate levels, and thriving at work not only affects job satisfaction directly, but also indirectly through organizational commitment. This study suggest that health policymakers should promote job satisfaction among PHWs through relative inventions aiming to improve their thriving at work and organizational commitment.

Strengths and limitations of this study

- The structural equation model was used to analyze the association between thriving at work, organizational commitment, and job satisfaction.
- The covariates, including participants' gender, age, years of working, and professional level are adjusted in the statistical analysis.
- Only primary PHWs in the eastern region of China were included in the study.
- The cross-sectional nature of this study cannot deduce the causal relationships between three variables.
- This study focused on the group of primary public health workers who combating the pandemic in frontline.

1.Introduction

In early 2020, an epidemic called COVID-19 suddenly swept the world, now causing millions of infections and deaths worldwide. WHO identified the outbreak as a Public Health Emergency of International Concern (PHEIC), which poses a huge threat to people's health and the global economy[1, 2]. Being the least medical organizations, China's primary healthcare units played an important role in the battle against COVID-19 in the frontline of defense[3].

For peer review only

Public health is a significant part of China's healthcare service system and an indispensable force in improving the health of community residents. Primary PHWs are responsible for the detection, prevention and control of various infectious diseases, occupational health examination, planned immunization and epidemiological survey[4, 5]. As the essential gatekeepers in the epidemic control, since the outbreak, primary PHWs have not only completed heavy workloads, but also risked being infected while working on the frontline[6, 7]. They are in a state of chronic psychological stress and gradually experience emotional exhaustion of their profession. In addition, their personal physical, energetic, and motivational strengths are in a state of increasingly overload, which have negative affect on their job satisfaction and thriving at work[8].

Job satisfaction is the subjective psychological state of employees' physiological and psychological perceptions of the work environment and the work itself[9]. Vladica's empirical study subdivided job satisfaction into internal and external satisfaction[10]. Nowadays, job satisfaction is used as the best predictor to measure employees' self-fulfillment of their professional values[11,12]. Unlike the development of large hospitals, primary care units in China are faced with multiple challenges, such as smaller platform with limited opportunities, employee’s lower salary, and weaker medical service delivery capacity[13]. While PHWs have not received highly attention for a long time, compared with physicians and nurses. This is why our study focus on the current status of job satisfaction, thriving at work of primary PHWs with their occupation and its environment.

Thriving at work as a positive psychological state characterized by a joint sense of vitality and learning, that ultimately contribute to the flourishing development of the organization according to the Spreitzer[14]. A socially embedded model of thriving at work suggests vitality represents the hedonic component of well-being, whereas learning, as a means of realizing one’s potential, which indicates job satisfaction is an external display of thriving at work[15]. Thriving at work is an crucial factor influencing the healthcare providers’ personal career development and planning, accordingly contributing to their professional fulfillment and maintaining health[16]. Zhao’s study

For peer review only

pointed out that healthcare providers in China who lacked thriving at work often led to low job satisfaction, which affected their work enthusiasm and stability[17]. It indicates a highly positive correlation between job satisfaction and thriving at work, both of which will be directly related to the level of quality of healthcare services[5, 18].

Organizational commitment usually refers to the relative strength of an individual's identification with and involvement in a particular organization[19]. Meyer and Allen argued that organizational commitment as a psychological phenomenon which encourages employees to stay with the organization and participate wholeheartedly in its work as their "side-bet", and regard it as multiple components that consists of three components: affective, continuance, and normative commitment[20]. The level of organizational commitment increases with intrinsic value, which is another key expression of thriving at work. A recent study by Kleine have confirmed a positive relationship between thriving at work and organizational commitment (RC=0.63)[21]. A study concentrated on the group of nurses noted that organizational commitment was a significant positive predictor of their job satisfaction[22]. In addition, researchers confirm that the each component of organizational commitment are the critical determinants that affect individual’s job satisfaction and life satisfaction[23, 24].

In the field of occupational research and medical practice, the job demands-resources (JD-R) model proposed by Bakker recently attracted attention with regard to creating organizational culture, improving employee’s job satisfaction, and reducing turnover in pandemic[25]. Job demands usually refer to the extent to which the duties of the job require employees to exert their own somatic, social or psychological (cognitive and emotional) efforts, such as workload, emotional demands and hostile work environment. Job resources, on the other hand, consist of a set of components that are beneficial or supportive to the employee, mainly in terms of intrinsic motivation, social praise or organizational belonging[26]. The JD-R model has been tested and validated in a large sample of different countries and industries[27-29]. We consider the increased workload and physical and mental exhaustion of primary PHWs during an epidemic as high job demands, and employees' organizational

For peer review only

commitment and perceived sense of thriving as job resources. Job demands and job resources interact mutually and are reflected in the job satisfaction which is the main outcome. Therefore, the model needs to be used to further explore what factors of job demand (resources) are associated with job satisfaction.

It is known that job satisfaction, thriving at work and organizational commitment are the key factors which would affect employee's career stability that the individual has a stable job for a certain period of time and does not have the idea of leaving easily[30]. However, the inherent influence mechanism among the three variables of PHWs is still unclear. And most existing studies have explored the job satisfaction of physicians or nurses in large hospitals, with less attention paid to the public health personnel group. Therefore, this study is equipped with some scientific research value for human resource in the field of PHWs. Therefore, the first aim of this study was to assess the current statues of thriving at work and job satisfaction among primary PHWs during the COVID-19 pandemic; the second aim was to construct SEM for these three variables among primary PHWs, empirically analyze their quantitative relationships, as well as give suggestions on the relevant policies and healthcare practice, because it's crucial to stabilize the workforce of primary PHWs through improving their job satisfaction. Meanwhile, we hypothesized that (1) thriving at work and organizational commitment are protective factors for job satisfaction among primary PHWs, and (2) thriving at work would indirectly and directly affect job satisfaction through organizational commitment.

2.Methods

2.1 Participants of the study

This study was a cross-sectional study. A convenience sampling method was used to survey 601 primary PHWs in 20 primary care units (community health service centers or rural health centers) in northern provinces of China which are less developed regions from May to October 2020.

Inclusion criteria for participants:(1) years of work in primary care units ≥ 1

For peer review only

year; (2) having participated in front-line work against the COVID-19 epidemic for more than 1 month; (3) their professional qualification is the licensed registered PHWs. Exclusion criteria were: (1) interns and trainees; (2) those whose work sites were non-primary organizations; (3) physicians or nurses. A total of 650 questionnaires were distributed in this survey, and 601 valid questionnaires were finally returned, with a valid return rate of 92.46%.

2.2 Measures

2.2.1 Socio-demographic information questionnaire

After referring to previous literature and expert consultation, we identified the following demographic information as the study variables: including the gender, age, education background, types of working units (community healthcare service center, township/village health center), years of working and professional levels of the participants.

2.2.2 Minnesota Satisfaction Questionnaire

The Minnesota Satisfaction Questionnaire (MSQ), a short form scale developed by Weiss, Dawis, England & Lofquist, which used to measure employee’s job satisfaction, contains two dimensions: internal and external satisfaction[31]. The MSQ has 20 items and is scored on a 5-point Likert scale, with "very dissatisfied", "dissatisfied", "fair", "satisfied", "very satisfied" and "satisfied" respectively. The higher the score, the higher the satisfaction level of primary PHWs with their work. Previous studies focused on the healthcare providers have shown that the Cronbach’s alpha value of this scale is 0.87-0.92[32, 33].

2.2.3 Organizational commitment questionnaire

The Organizational commitment questionnaire (QCQ), developed by Allen and Meyer[34] was used in this study to measure the organizational commitment of primary PHWs. It contains three dimensions: affective commitment, normative commitment, and sustaining commitment, and each dimension includes six items. All items are

For peer review only

measured on a Likert 5-point scale from 1 (strongly disagree) to 5 (strongly agree). The Cronbach alpha value for each dimension of the scale was 0.928, 0.802, and 0.859[35]. The total scale score was the sum of the items and ranged from 18 to 90, with higher scores representing higher levels of employee's organizational commitment[36].

2.2.4 Thriving at work scale

The Thriving at work scale developed by Porath[37] was used, which has 10 items and measures two dimensions of thriving at work, vitality and learning, each containing 5 items. This is a Likert 5 scale of "1 to 5" represents "very unlikely ~ very likely", respectively. The higher the score, the higher the sense of thriving at work of the participants. The Chinese version of the questionnaire has been shown to have good reliability and validity in the Chinese teachers and nurses population[38, 39].

2.3 Data collection

This study was an anonymous and written survey, all the data obtained was confidentiality promised. Rigorous training and assessment of the investigators were conducted before the survey began, and the participants all signed an informed consent priority to the study. The paper questionnaires were distributed together by the investigators in cooperation with the leaders of the units or organizations, and the participants were explained using an instruction. The questionnaires were carefully checked by the investigators after the participants had completed them and were collected in time. This study was approved by the Ethics Committee of Shandong University in 15th July, 2020, China, and the approval No. is 2020-R01-23.

2.4 Statistical analysis

SPSS version 21.0 was used to analyze the data descriptively. t-test and one-way ANOVA were used to compare the differences between variables on the socio-demographic characteristics of the study population. Pearson correlation analysis was used to clarify the correlation between organizational commitment, job satisfaction and thriving at work among primary PHWs, with a p-value smaller than 0.05 being

For peer review only

considered a statistically significant difference. Based on the correlation analysis, AMOS 26.0 was used to develop a SEM to analyze the inherent relationship between the three variables. The path coefficients between the latent variables were tested using path analysis, and the parameter estimation was performed using the great likelihood method, assuming that the significance of the mediating effect of organizational commitment in the model was calculated using Bootstrap analysis with 2000 samples with the test level set at α=0.05, and if the 95% confidence interval of the standardized path coefficients did not contain 0, indicates the mediating effect was significant. The SEM can be considered well-fitted when all path coefficients satisfy the criteria of Hu and Bentler's study[40].

3. Results

3.1 General Information

A total of 650 primary PHWs who had participated in the frontline of epidemic fighting were surveyed in 2020. The average age of the participants was (36.61±8.95) years old, with the majority being female (82.9%), and the education background of all participants was mainly college or above (94.0%), the average working years were (14.31±6.21) years, and their professional levels which is a comprehensive reflection of their work experience and work ability are mainly in junior level (72.3%). For the three main scales involved in this study, we did the relevant Cronbach coefficient tests, and the results were as follows: (1) the Cronbach's alpha value of Minnesota Satisfaction Questionnaire was 0.919; (2) the Cronbach's alpha value of organizational commitment scale in this study was 0.917; (3) the overall Cronbach's alpha value of Thriving at work scale was 0.89, and the Cronbach′s α alpha for the vitality and learning dimensions were 0.87 and 0.89, respectively.

3.2 Analysis of common method bias test

In this study, common method bias test was conducted using Harman's one-way test and exploratory factor analysis was performed without rotation. The results

For peer review only

showed that there were 14 factors with characteristic roots greater than 1. The variance rate explained by the first factor of job satisfaction was 36.73%, which was less than the critical value of 40%, indicating that there was no serious problem of common method bias in this study[41].

3.3 Demographic differences in thriving at work and job satisfaction scores

The results showed that for primary PHWs, there were statistical differences in thriving at work on two demographic variables: education, and years of working. the PHWs who obtained lower education degree have higher thriving at work, additionally, the lowest level of thriving at work was observed in the group of the year of working more than 20 years.

There were statistical differences in job satisfaction among primary PHWs on three demographic variables: gender, education and years of working. Female PHWs had higher scores than males in the job satisfaction; PHWs with higher years of working had higher level of job satisfaction; PHWs with better education background had lower job satisfaction, as detailed in **Table 1**.

Table 1 Demographic differences in Thriving at Work and Job Satisfaction among primary PHWs

Variables	Scores of T W		Scores of JS	
	scores	*t/F* value	scores	*t/F* value
**Gender**		0.762		1.825**
Male	3.13±0.70		3.91±0.80	
Female	3.18±0.64		4.07±0.67	
**Age**		0.126		0.066
＜30 years old	3.16±0.74		3.04±0.74	
30-45 years old	3.19±0.59		3.06±0.67	
＞45 years old	3.16±0.63		3.05±0.68	
**Years of working**		0.267*		1.873*
＜10 years	3.15±0.69		3.05±0.72	
10 -20 years	3.20±0.61		3.03±0.68	
＞20 years	3.17±0.67		3.06±0.66	

For peer review only - http://bmjopen.bmj.com/site/about/guidelines.xhtml

**Educational background**		0.396*		0.785**
College and below	3.16±0.54		3.08±0.57	
University Undergraduate	3.12±0.65		3.02±0.68	
Postgraduate and above	3.05±0.61		3.96±0.46	
**Professional level**		0.311		0.655
Junior	3.05±0.72		3.05±0.72	
Intermediate	3.03±0.68		3.03±0.68	
Advanced	3.04±0.66		3.06±0.66	

Abbreviations: TW, thriving at work; OC, organizational commitment; JS, job satisfaction.

Note: $^*P< 0.05$, $^{**}P< 0.01$.

3.4 Correlation between organizational commitment, job satisfaction and thriving at work among primary PHWs

The results showed that the mean score of thriving at work of the 601 surveyed primary PHWs was (3.17±0.65). The mean score of their organizational commitment score was (2.93±0.73). and the mean score of their job satisfaction score was (3.05±0.69). Pearson's correlation analysis revealed that the thriving at work was positively correlated with organizational commitment (r=0.715, P<0.001) and highly positively correlated with job satisfaction (r=0.804, P<0.001); furthermore, there was a highly positive correlation between organizational commitment and job satisfaction (r=0.848, P<0.001), see **Table 2**.

Table 2 Correlation between thriving at work, organizational commitment and job satisfaction among primary PHWs

Variables	Mean ± SD	TW	OC	JS
TW	3.17±0.65	1	—	—
OC	2.93±0.73	0.715**	1	—
JS	3.05±0.69	0.804**	0.848**	1

Abbreviations: TW, thriving at work, OC, organizational commitment, JS, job satisfaction

Note: $^*P< 0.05$, $^{**}P< 0.01$.

For peer review only

3.5 SEM of organizational commitment, job satisfaction, and thriving at work among primary PHWs

In order to investigate the influence mechanism among the variables in depth, after controlling for the gender, age, education background, and years of working of the participants. A SEM was constructed with thriving at work as the independent variable, organizational commitment as the mediating variable, and job satisfaction as the dependent variable. AMOS 26.0 software was used to conduct the validation analysis of the hypothesized model, and the results showed that each path coefficient was statistically significant ($p<0.001$), as shown in **Figure 1**. The model fit statistics were: chi-squared degrees of freedom (χ^2/df) =3.416, GFI=0.982, AGFI=0.953, NFI=0.993, CFI=0.995, IFI=0.995, and RMSEA=0.063 (95% confidence interval 0.042,0.086), respectively. All the above fit indices are within the acceptable range and the model fits well, as shown in **Table 3**.

Abbreviations: CC, continuance commitment; NC, normative commitment; AC, affective commitment; IS, internal satisfaction; ES, external satisfaction.

Figure 1 Structural equation model of primary PHWs' thriving at work, organizational commitment, job satisfaction

For peer review only

Table 3 Index Evaluation System and Fitting Results of Structural Equation Model Adaptability

Fit Index	χ^2/df	GFI	AGFI	RMSEA	IFI	CFI	NFI
Theoretical Model	＜3	＞0.9	＞0.9	＜0.08	＞0.9	＞0.9	＞0.9
Regression criterion	3.416	0.982	0.953	0.063	0.995	0.995	0.993

Abbreviations: *df*, degree of freedom; GFI, goodness-of-fit index; AGFI, adjusted goodness-

of-fit index; RMSEA, root-mean-square error of approximation; IFI: Incremental Fit Index; CFI, comparative fit index; NFI, normed fit index.

Bootstrap method was used to test the significance of the mediating role of organizational commitment between thriving at work and job satisfaction. Repeated samples were taken 2000 times and 95% confidence intervals were calculated. The results showed that the standardized effect value of the mediating effect of organizational commitment between thriving at work and job satisfaction was 0.422 with 95% *CI* (0.276,0.561); the standardized effect value of the total effect was 0.867 with 95% *CI* (0.781,0.920), both of which did not contain 0, indicating that the differences in all effect paths were statistically significant. The calculated mediated effect size was 48.7% of the total effect size, and the specific path coefficients of the model are shown in **Table 4**.

Table 4 Pathway coefficients of structural equation model (n=601)

Pathways	Std. estimate	95% *CI*	SE	*P*-value
**Total effect**				
	0.867	(0.781,0.920)		0.002**
**Direct effect**				
TW→JS	0.445	(0.269,0.640)	0.098	0.001**
TW→OC	0.765	(0.662,0.844)	0.046	＜0.01**
OC→JS	0.552	(0.364,0.726)	0.096	0.001**
**Indirect effect**				
TW→OC→JS	0.422	(0.276,0.561)	0.070	0.001**

Abbreviations: TW, thriving at work; OC, organizational commitment; JS, job satisfaction; Std. Estimate, Standardized estimate; CI, Confidence interval; SE, Standard error.

Note: $^{*}P< 0.05$, $^{**}P< 0.01$.

4. Discussion

Based on the JD-R model, this study found that during the COVID-19 pandemic,

For peer review only

primary PHWs in China had a moderate level of job satisfaction. Primary PHWs with adequate thriving at work are more likely to exhibit higher job satisfaction. Besides, thriving at work can also indirectly influence PHWs' job satisfaction through organizational commitment.

The current status of thriving at work and job satisfaction among primary PHWs

Job satisfaction is one of the indispensable indicator of the quality of work done by primary PHWs, which has an important impact on their physical and mental health development[42]. However, during the pandemic in China, when small-scale outbreaks occurred in some area, primary PHWs are on the frontline of the fight against the epidemic and are required to respond quickly to their areas of responsibility[43]. Consequently, they are exposed to greater workload, physical injuries and mental stress caused by higher work demands, with insufficient work resources, which seriously deteriorate their job satisfaction[44]. The results showed that the thriving at work score of primary PHWs was 3.17±0.65. Besides, the score of job satisfaction was 3.05±0.69, which was lower than the score of PHWs in Nigeria and Iran[45, 46]. Meanwhile, the results of the ANOVA analysis showed that there were statistically significant differences in the job satisfaction scores of primary PHWs in variables of gender, years of working, and education background. That is, primary PHWs who are male, with higher education, fewer years of working experience, are at a relatively lower level of job satisfaction. The potential explanations could be the fact that grassroots position offer fewer promotion and self-development opportunities, inadequate salary and insufficient supportive measures, resulting in primary PHWs with master degree or higher cannot give full play to their knowledge into the healthcare practice, thus limiting their long-term career development, as well as causing lower job satisfaction for them[47]. Besides, when employees have lower years of working experience, their coping ability is still in the ascending stage, it is not possible to be very proficient in work operations and has limited access to work resources, especially in the heavy epidemic period, which to a certain extent reduces

For peer review only

their job satisfaction[48].

Correlation and SEM between organizational commitment, thriving at work, and job satisfaction among primary PHWs

Previous studies have shown a positive relationship between organizational commitment and their job satisfaction among various industries, while such relationship has not been confirmed among primary PHWs[49]. Firstly, The results of Pearson analysis showed that thriving at work have a significantly positive correlation with job satisfaction (β=0.804, P<0.01), the integrated model of personal growth of 'thriving at work' suggests that a key output of thriving at work is the indicator of job satisfaction[50]. A meta-analysis that included 21,739 subjects has found that teachers with high level of thriving at work would exhibit greater job satisfaction[51]. In addition, thriving at work is one of the significant factors of the work resource for the healthcare occupation and health-related organizations. Individual's thriving focuses on two domains, vitality and learning, in daily work[52]. Primary PHWs can enhance learning through various types of training, to create a positive work atmosphere that is highly beneficial to both individuals and healthcare organizations. Moreover, vitality can help individuals adapt creatively to the difficulties of their at some situation, especially during the risky and challenging COVID-19 pandemic[53]. Secondly, the organizational commitment kept a highly positive relationship with job satisfaction(β =0.848, P<0.01). PHWs with high organizational commitment have a deep attachment to their healthcare units and a strong sense of responsibility and mission for their jobs. The correlation between three components of organizational commitment and job satisfaction can be reflected as follows: if the primary PHWs exhibits higher affective commitment, they may have greater identification with the organization's goals, values and culture, and higher satisfaction with their job[54]. Besides, continuance commitment facilitates primary PHWs loyalty to the organization, which in turn enhances their satisfaction. And the normative commitment ensures the primary PHWs believe that they have a great responsibility

For peer review only

to the organization, the greater the psychological satisfaction that active work can bring to the individual, and the higher their job satisfaction[55]. Finally, there is a positive relationship between thriving and organizational commitment, and thriving on the content, atmosphere, and nature of their work promotes employees' attachment to the organization, they are more likely to derive higher satisfaction from their work[56].

The results of the SEM in this study also confirmed that organizational commitment partially mediated the relationship between thriving at work and job satisfaction, with a mediating effect size of -0.35, accounting for 57.38% of the total effect, indicating that organizational commitment serve as a significant factor affecting primary PHWs' level of job satisfaction. This is consistent with the result of Malgorzata's study, but his study participants were cross-cultural workers[57]. So the new relationship between thriving at work, organizational commitment and job satisfaction confirmed in this study is the first validation in a group of Chinese healthcare providers. Furthermore, Bakker's study supports that organizational commitment is the result of a combination of many different job demands and job resources. Positive organizational commitment can have an impact on job satisfaction[58]. According to psychological contract theory, the cognitive structure of individuals determines how public health workers view their relationship with the organization. This is considered to be their belief system, the obligations that exist between them and their work healthcare organization[59]. Therefore, the mechanisms underlying the relationship indicates that thriving at work is a state of need and motivation that is critical to the internal psyche of the individual and that is easily activated and motivated by situational factors. a theoretical model of thriving at work developed by Spreitzer and colleagues[60], which suggests that when employees feel energized and full of learning in the workplace, they are energized and willing to invest more time, energy, and effort in their work, resulting in high level of thriving at work. This will further stimulate their identification with their organizations (i.e., affective commitment), which will lead to a higher level of centripetal force toward their careers and the groups they work with (i.e., continuance commitment), and a

greater willingness to engage in organizational activities and construction out of their own strong sense of responsibility and mission (i.e., normative commitment)[61]. Additionally, employees' commitment to the organization inevitably affects employees' attitudes or feelings toward work, which in turn affects their job satisfaction. Positive work can bring psychological satisfaction to the individual, so the higher the employee's job satisfaction, i.e., higher satisfaction with the job content, the nature of the job, and the atmosphere of the job.

This study explored the intrinsic association between thriving at work and job satisfaction by constructing a SEM with organizational commitment as a mediating variable. Thriving has been proved can promote career sustainability among primary PHWs and can have a direct impact on their job satisfaction, as well as an indirect impact on their job satisfaction by increasing the level of organizational commitment. This study provides a theoretical basis for better stabilizing the talent pool of primary PHWs in China, improving the service quality of primary public health and medical care in China, and optimizing the allocation of primary medical resources.

Implications on policies or practices

The suggestions on the policies and practices can be made from the perspective of health policymakers, as well as from the management of leaders. Firstly, the health policymakers are advised to refer to the way of magnet hospitals, although the magnet hospital originally was applied to nursing for initiatives to ameliorate the environment in which nurses practice. Specifically, policymakers need to focus on providing a platform for staff's career development, as well as providing supportive continuing education. Establishing a scientific and reasonable salary system and creating a positive organization culture is conducive to mobilizing and motivating primary PHWs, thus improving their job satisfaction. Secondly, for the leadership of employers, the leaders should fully explore the potential advantages of primary PHWs in thriving at work, establish a relationship of mutual trust with their subordinates, respond positively to their opinions and suggestions, and cultivate their sense of organizational identity. Consequently, primary PHWs are more willing to integrate

For peer review only

the organization's development goals into their personal values and ideals, thus generating a higher level of organizational commitment, which is an intrinsic motivation for their proactive efforts.

Study limitations

Firstly, participants in this study may have had recall bias when completing the scales. Secondly, this study was a multicenter study conducted in primary healthcare units in northern China. Therefore, caution should be exercised when extending the results of this study to other countries, especially developed countries. Finally, because this study was a cross-sectional study, there was no strong causality. Future longitudinal studies with larger samples and incorporating more variables are needed to validate the results of this study.

5. Conclusion

This study reveals that Chinese primary PHWs faced high job demand and limited job resources, leading to their poor thriving and job satisfaction. Meanwhile SEM results found that thriving at work has a direct contribution to job satisfaction, while organizational commitment as a mediating variable can also increase job satisfaction. Therefore, in the background of COVID-19 pandemic, health policymakers should pay high attention to this group of primary PHWs, as well as enhance their thriving and organizational commitment by regulating their job demands and providing sufficient job resources to effectively activate their job satisfaction and fulfillment, which is essential to maintain the career stability of primary PHWs.

Author Contributions

Conceived and designed the experiments: Mo Yi, Di Jiang, Ou Chen.

Performed the experiments: Yuanmin Jia, Jingjing Wang, Lei Guo, Zeyi Zhang.

For peer review only

Analyzed the data: Mo Yi, Yuanmin Jia, Mo Yi, Di Jiang

Contributed reagents/materials/analysis tools: Yuanmin Jia, Baosheng Zhao.

Wrote the manuscript: Mo Yi.

Mo Yi and Di Jiang are co-first authors of the article

Acknowledgments

We are particularly grateful to the coordinators who assisted us, especially all the primary PHWs who participated in this study and supported the study during the busy period of epidemic prevention and control.

Funding

This study was supported by The Natural Science Foundation of China (82172543); The Natural Science Foundation of Shandong Province (ZR2020MH006); The Key Research and Development Program of Shandong Province (2019GSF108198).

Disclosure

The authors report no conflicts of interest in this work.

Data availability statement

The data or models generated or used during the study are available from the corresponding author by request.

Patient and Public Involvement

No patient involved

Ethics statement

The study was approved by the ethic board of School of Nursing and

For peer review only

Rehabilitation, Shandong University, China. All participants provided written informed consent.

References:

1. Lancet T. COVID-19: too little, too late? *Lancet (London, England).* 2020(No.10226):755.

2. Liu C, Zhao Y, Okwan-Duodu D, Basho R, Cui X. COVID-19 in cancer patients: risk, clinical features, and management. *Cancer Biol Med.* 2020(No.3):519-527.

3. Wang H, Tang C, Zhao S, Meng Q, Liu X. Job Satisfaction among Health-Care Staff in Township Health Centers in Rural China: Results from a Latent Class Analysis. *Int J Environ Res Public Health.* 2017;14(10).

4. Shi LSL, Song KSK, Rane SRS, Sun XSX, Li HLH, Meng QMQ. Factors associated with job satisfaction by Chinese primary care providers. *Prim Health Care Res.* 2014(No.1):46-57.

5. Zhou J, Lee DM, Yiqi J, Xuan J. Thriving at work: A mentoring-moderated process linking task identity and autonomy to job satisfaction. *J Vocat Behav.* 2020;118(C).

6. Li J, Xu J, Zhou H, et al. Working conditions and health status of 6,317 front line public health workers across five provinces in China during the COVID-19 epidemic: a cross-sectional study. *Bmc Public Health.* 2021;21(1):106.

7. Li J, Xu J, Zhou H, et al. Working conditions and health status of 6,317 front line public health workers across five provinces in China during the COVID-19 epidemic: a cross-sectional study. *Bmc Public Health.* 2021;21(1).

8. Jing Zhang YWJX. Prevalence of mental health problems and associated factors among front-line public health workers during the COVID-19 pandemic in China: an effort-reward imbalance model-informed study. *BMC psychology.* 2021(No.1):55.

9. Kuo HT, Lin KC, Li IC. The mediating effects of job satisfaction on turnover intention for long-term care nurses in Taiwan. *J Nurs Manag.* 2014;22(2):225-233.

10. Veličković VMA, Višnjić AAB, Jović SA, et al. Organizational commitment and job satisfaction among nurses in Serbia: A factor analysis(Article). *Nurs Outlook.* 2014(No.6):415-427.

11. Eacute P, Maximilian, Haefner N, Golden T. Clarifying CSE on Job Performance: The Roles of Innovative Work Behavior & Transformational Leaders. *Academy of Management Annual Meeting*

For peer review only

Proceedings. 2018(No.1):1.

12. Zhou J, Lee DM, Yiqi J, Xuan J. Thriving at work: A mentoring-moderated process linking task identity and autonomy to job satisfaction. *J Vocat Behav.* 2020;118(C).

13. Wang LWL, Wang ZWZ, Ma QMQ, Fang GFG, Yang JYJ. The development and reform of public health in China from 1949 to 2019. *Globalization & Health.* 2019(No.1):45.

14. Spreitzer GCFP, Sutcliffe KCFP, Dutton JCFP, Sonenshein SCFP, Grant AMCF. A Socially Embedded Model of Thriving at Work. *Organ Sci.* 2005(NO.5):537-549.

15. Abid G, Khan B, Hong MC. Thriving at Work: How Fairness Perception Matters for Employee's Thriving and Job Satisfaction. *Academy of Management Annual Meeting Proceedings.* 2016(No.1):1.

16. Abid GGAH, Zahra IIZY, Ahmed ADNE. Mediated Mechanism of Thriving at Work between Perceived Organization Support, Innovative Work Behavior and Turnover Intention. *Pakistan Journal of Commerce & Social Sciences.* 2015(No.3):982-998.

17. Zhao SH, Shi Y, Sun ZN, et al. Impact of workplace violence against nurses' thriving at work, job satisfaction and turnover intention: A cross-sectional study. *J Clin Nurs.* 2018;27(13-14):2620-2632.

18. Zhang S, Shi Y, Liu B, et al. Job demands and resources and their relationship with satisfaction and thriving at work in a sample of Chinese doctors: a cross-sectional study. *Bmj Open.* 2021;11(11).

19. Güleryüz GHUD, Güney SHUD, Aydın EMHU, Aşan ÖHUD. The mediating effect of job satisfaction between emotional intelligence and organisational commitment of nurses: A questionnaire survey. *Int J Nurs Stud.* 2008(No.11):1625-1635.

20. Meyer JP, Allen NJ. A three-component conceptualization of organizational commitment. *Hum Resour Manage R.* 1991(NO.1):61-89.

21. Kleine AKAA, Rudolph CWA, Zacher HAHZ. Thriving at work: A meta‐analysis. *J Organ Behav.* 2019(No.9-10):973-999.

22. Aharon AA, Madjar B, Kagan I. Organizational commitment and quality of life at work among public health nurses in Israel. *Public Health Nurs.* 2019(No.4):534-540.

23. Culibrk JCJ, Delic MDM, Mitrovic SMS, Culibrk DCD. Job Satisfaction, Organizational Commitment and Job Involvement: The Mediating Role of Job Involvement. *Front Psychol.* 2018:132.

24. Eric G Lambert RTSE. The Association of Organizational Justice with Job Satisfaction and Organizational Commitment among Nigerian Correctional Staff. *Int J Offender Ther.* 2021(No.2-3):180-204.

For peer review only

25. Demerouti E, Bakker AB. The Job Demands-Resources model: challenges for future research. *SA Journal of Industrial Psychology.* 2011;37(2):1-9.

26. Zis P, Anagnostopoulos F, Sykioti P. Burnout in medical residents: a study based on the job demands-resources model. *TheScientificWorldJournal.* 2014;2014:673279.

27. Bakker AB, Demerouti E, Taris TW, Schaufeli WB, Schreurs PJG. A multigroup analysis of the job demands-resources model in four home care organizations. *Int J Stress Manage.* 2003;10(1).

28. Zeng W, Ma S, Callan VJ, Wu L. Exploring the doctor-patient relationship as a challenge job demand: application of the job demands-resources model in a Chinese public hospital. *Psychology, health & medicine.* 2021.

29. Sabine K, Andreas R. A job demands-resources perspective on salespersons' market intelligence activities in new product development. *Journal of Personal Selling & Sales Management.* 2016;36(1).

30. Howes LMA, Goodman-Delahunty JB. Predicting Career Stability and Mobility: Embeddedness and Boundarylessness(Article). *J Career Dev.* 2015(No.3):244-259.

31. Weiss DJ, Dawis RVE, G. W. And Lofquist LH. *Manual for the Minnesota Satisfaction Questionnaire*. Vol. 22 ed. Industrial Relations Center: Minnesota Studies in Vocational Rehabilitation, Minneapolis: University of Minnesota; 1967.

32. Li N, Zhang L, Xiao G, Chen J, Lu Q. The relationship between workplace violence, job satisfaction and turnover intention in emergency nurses. *Int Emerg Nurs.* 2019:50-55.

33. Zhang X, Bian L, Bai X, et al. The influence of job satisfaction, resilience and work engagement on turnover intention among village doctors in China: a cross-sectional study. *Bmc Health Serv Res.* 2020;20(1):283.

34. Allen NJUW, Meyer JP. Affective, continuance, and normative commitment to the organization: An examination of construct validity. *J Vocat Behav.* 1996(No.3):252-276.

35. NGUYEN HM, NGO TT. Psychological Capital, Organizational Commitment and Job Performance: A Case in Vietnam. *The Journal of Asian Finance, Economics and Business.* 2020:269-278.

36. Lin WQA, Yuan LXB, Kuang SYB, et al. Work engagement as a mediator between organizational commitment and job satisfaction among community health-care workers in China: a cross-sectional study. *Psychology, Health and Medicine.* 2020(No.6):666-674.

37. Porath CA, Spreitzer GASU, Gibson CA, Garnett FGA. Thriving at work: Toward its

For peer review only

measurement, construct validation, and theoretical refinement. *J Organ Behav.* 2012(No.2):250-275.

38. Yi Yang XL. The Impact of Challenge and Hindrance Stressors on Thriving at Work Double Mediation Based on Affect and Motivation. *Front Psychol.* 2021:613871.

39. X Zhu WKSS. A causal model of thriving at work in Chinese nurses. *Int Nurs Rev.* 2021:1.

40. Hu L, Bentler PM. Fit indices in covariance structure modeling: Sensitivity to underparameterized model misspecification. *Psychol Methods.* 1998(No.4):424-453.

41. Doty DHSU, Glick WH. Common Methods Bias: Does Common Methods Variance Really Bias Results? *Organ Res Methods.* 1998(NO.4):374-406.

42. Warr P, Inceoglu I. Job Engagement, Job Satisfaction, and Contrasting Associations With Person-Job Fit.[Article]. *J Occup Health Psych.* 2012(No.2):129-138.

43. Huang J, Liu F, Teng Z, et al. Care for the Psychological Status of Frontline Medical Staff Fighting Against Coronavirus Disease 2019 (COVID-19). *Clin Infect Dis.* 2020;71(12):3268-3269.

44. She R, Wang X, Zhang Z, et al. Mental Health Help-Seeking and Associated Factors Among Public Health Workers During the COVID-19 Outbreak in China. *Front Public Health.* 2021;9:622677.

45. Adeniji AA, Osibanjo AO, Iruonagbe TC, Olawande T, Ibidunni AS, Olokundun MA. From job satisfaction to organizational commitment: The mediating influence of perceived treatment of diversity among nigeria’ s public healthcare employees(Article). *Open Access Macedonian Journal of Medical Sciences.* 2019(No.12):2031-2035.

46. Sarabi RE, Javanmard R, Shahrbabaki PM. Study of burnout syndrome, job satisfaction and related factors among health care workers in rural areas of Southeastern Iran. *AIMS Public Health.* 2020(No.1):158-168.

47. Li L, Hu H, Zhou H, et al. Work stress, work motivation and their effects on job satisfaction in community health workers: a cross-sectional survey in China. *Bmj Open.* 2014;4(6):e4897.

48. Lu YLY, Hu XHX, Huang XHX, et al. Job satisfaction and associated factors among healthcare staff: a cross-sectional study in Guangdong Province, China. *Bmj Open.* 2016(No.7):e11388.

49. Ghulam A, Francoise C, Saira A, Tehmina Q. Contextual Factors and Organizational Commitment: Examining the Mediating Role of Thriving at Work. *Sustainability-Basel.* 2019;11(17).

50. Spreitzer GM, Porath CL. Self-Determination as a Nutriment for Thriving: Building an Integrative Model of Human Growth at Work. *The Oxford Handbook of Work Engagement, Motivation, and Self-Determination Theory.* 2014.

For peer review only

51. Kleine AKAA, Rudolph CWA, Zacher HAHZ. Thriving at work: A meta - analysis. *J Organ Behav.* 2019(No.9-10):973-999.

52. Danping Liu SZYW. The Antecedents of Thriving at Work: A Meta-Analytic Review. *Front Psychol.* 2021:659072.

53. Ding YDY, Qu JQJ, Yu XYX, Wang SWS. The Mediating Effects of Burnout on the Relationship between Anxiety Symptoms and Occupational Stress among Community Healthcare Workers in China: A Cross-Sectional Study. *Plos One.* 2014(No.9):e107130.

54. Yang F, Chang C. Emotional labour, job satisfaction and organizational commitment amongst clinical nurses: a questionnaire survey. *Int J Nurs Stud.* 2008;45(6).

55. Darwish AY. Job Satisfaction as a Mediator of the Relationship Between Job Stressors and Affective, Continuance, and Normative Commitment: A Path Analytical Approach. *Int J Stress Manage.* 2002;9(2).

56. Harris O, Jacobsen P, Searle-Barnes R, Wilkinson-Tough M. Surviving and thriving - a mixed-methods study of staff experiences of occupational wellbeing in a psychiatric place of safety service. *J Ment Health.* 2020.

57. Rozkwitalska M, Basinska BA. THRIVING AND JOB SATISFACTION IN MULTICULTURAL ENVIRONMENTS OF MNCS. *SMART AND EFFICIENT ECONOMY: PREPARATION FOR THE FUTURE INNOVATIVE ECONOMY.* 2016:111-119.

58. Arnold BB, Marc VV, Despoina X. Beyond the Demand-Control Model: Thriving on High Job Demands and Resources. *J Pers Psychol.* 2010;9(1).

59. Ge J, He J, Liu Y, et al. Effects of effort-reward imbalance, job satisfaction, and work engagement on self-rated health among healthcare workers. *Bmc Public Health.* 2021;21(1).

60. Spreitzer G, Porath CL, Gibson CB. Toward human sustainability: How to enable more thriving at work. *Organ Dyn.* 2012;41(2SI):155-162.

61. Gielissen KA, Taylor EP, Vermette D, Doolittle B. Thriving among Primary Care Physicians: a Qualitative Study. *J Gen Intern Med.* 2021;36(12).

For peer review only

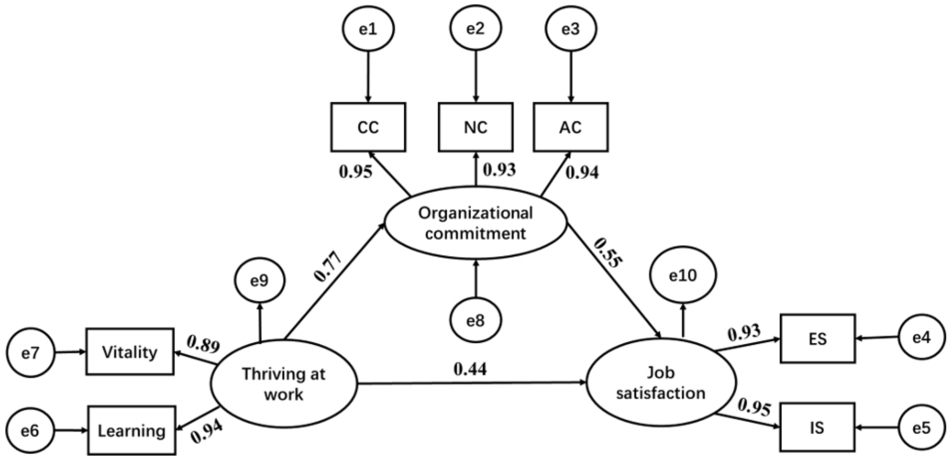

Structural equation model of primary PHWs' thriving at work, organizational commitment, job satisfaction

447x233mm (57 x 57 DPI)

STROBE Statement—Checklist of items that should be included in reports of ***cross-sectional studies***

	Item No	Recommendation	Page No
**Title and abstract**	1	(*a*) Indicate the study's design with a commonly used term in the title or the abstract	2
		(*b*) Provide in the abstract an informative and balanced summary of what was done and what was found	2
**Introduction**			
Background/rationale	2	Explain the scientific background and rationale for the investigation being reported	3
Objectives	3	State specific objectives, including any prespecified hypotheses	5
**Methods**			
Study design	4	Present key elements of study design early in the paper	5
Setting	5	Describe the setting, locations, and relevant dates, including periods of recruitment, exposure, follow-up, and data collection	5
Participants	6	(*a*) Give the eligibility criteria, and the sources and methods of selection of participants	5
Variables	7	Clearly define all outcomes, exposures, predictors, potential confounders, and effect modifiers. Give diagnostic criteria, if applicable	5,6
Data sources/ measurement	8*	For each variable of interest, give sources of data and details of methods of assessment (measurement). Describe comparability of assessment methods if there is more than one group	7
Bias	9	Describe any efforts to address potential sources of bias	7
Study size	10	Explain how the study size was arrived at	7
Quantitative variables	11	Explain how quantitative variables were handled in the analyses. If applicable, describe which groupings were chosen and why	7
Statistical methods	12	(*a*) Describe all statistical methods, including those used to control for confounding	8
		(*b*) Describe any methods used to examine subgroups and interactions	8
		(*c*) Explain how missing data were addressed	8
		(*d*) If applicable, describe analytical methods taking account of sampling strategy	8
		(*e*) Describe any sensitivity analyses	8
**Results**			
Participants	13*	(a) Report numbers of individuals at each stage of study—eg numbers potentially eligible, examined for eligibility, confirmed eligible, included in the study, completing follow-up, and analysed	9
		(b) Give reasons for non-participation at each stage	9
		(c) Consider use of a flow diagram	
Descriptive data	14*	(a) Give characteristics of study participants (eg demographic, clinical, social) and information on exposures and potential confounders	9
		(b) Indicate number of participants with missing data for each variable of interest	9
Outcome data	15*	Report numbers of outcome events or summary measures	10
Main results	16	(*a*) Give unadjusted estimates and, if applicable, confounder-adjusted estimates and their precision (eg, 95% confidence interval). Make clear which confounders were adjusted for and why they were included	10

For peer review only

		(*b*) Report category boundaries when continuous variables were categorized	11
		(*c*) If relevant, consider translating estimates of relative risk into absolute risk for a meaningful time period	11
Other analyses	17	Report other analyses done—eg analyses of subgroups and interactions, and sensitivity analyses	12,13
**Discussion**			
Key results	18	Summarise key results with reference to study objectives	13-17
Limitations	19	Discuss limitations of the study, taking into account sources of potential bias or imprecision. Discuss both direction and magnitude of any potential bias	17
Interpretation	20	Give a cautious overall interpretation of results considering objectives, limitations, multiplicity of analyses, results from similar studies, and other relevant evidence	17
Generalisability	21	Discuss the generalisability (external validity) of the study results	17
**Other information**			
Funding	22	Give the source of funding and the role of the funders for the present study and, if applicable, for the original study on which the present article is based	18

*Give information separately for exposed and unexposed groups.

Note: An Explanation and Elaboration article discusses each checklist item and gives methodological background and published examples of transparent reporting. The STROBE checklist is best used in conjunction with this article (freely available on the Web sites of PLoS Medicine at http://www.plosmedicine.org/, Annals of Internal Medicine at http://www.annals.org/, and Epidemiology at http://www.epidem.com/). Information on the STROBE Initiative is available at www.strobe-statement.org.

For peer review only

BMJ Open

Relationships among thriving at work, organizational commitment and job satisfaction among Chinese frontline primary public health workers during COVID-19 pandemic: a structural equation model analysis

Journal:	*BMJ Open*
Manuscript ID	bmjopen-2021-059032.R2
Article Type:	Original research
Date Submitted by the Author:	03-May-2022
Complete List of Authors:	Yi, Mo; Shandong University, Cheeloo College of Medicine, School of Nursing and Rehabilitation<br>Jiang, Di; Peking Union Medical College Hospital Eastern Branch, Department of Nursing<br>Wang, Jingjing ; Shandong University, Cheeloo College of Medicine, School of Nursing and Rehabilitation<br>Zhang, Zeyi ; Shandong University, Cheeloo College of Medicine, School of Nursing and Rehabilitation<br>Jia, Yuanmin; Shandong University, School of Nursing and Rehabilitation<br>Zhao, Baosheng; Shandong University, Cheeloo College of Medicine, School of Nursing and Rehabilitation<br>Guo, Lei; Shandong University, Cheeloo College of Medicine, Centre for Health Management and Policy Research; Shandong University, Cheeloo College of Medicine, School of Public Health<br>Chen, Ou ; Shandong University, Cheeloo College of Medicine, School of Nursing and Rehabilitation
**Primary Subject Heading**:	Public health
Secondary Subject Heading:	Health services research, Medical management, Occupational and environmental medicine, Infectious diseases
Keywords:	COVID-19, Health & safety < HEALTH SERVICES ADMINISTRATION & MANAGEMENT, Human resource management < HEALTH SERVICES ADMINISTRATION & MANAGEMENT, PUBLIC HEALTH

SCHOLARONE™ Manuscripts

I, the Submitting Author has the right to grant and does grant on behalf of all authors of the Work (as defined in the below author licence), an exclusive licence and/or a non-exclusive licence for contributions from authors who are: i) UK Crown employees; ii) where BMJ has agreed a CC-BY licence shall apply, and/or iii) in accordance with the terms applicable for US Federal Government officers or employees acting as part of their official duties; on a worldwide, perpetual, irrevocable, royalty-free basis to BMJ Publishing Group Ltd ("BMJ") its licensees and where the relevant Journal is co-owned by BMJ to the co-owners of the Journal, to publish the Work in this journal and any other BMJ products and to exploit all rights, as set out in our licence.

The Submitting Author accepts and understands that any supply made under these terms is made by BMJ to the Submitting Author unless you are acting as an employee on behalf of your employer or a postgraduate student of an affiliated institution which is paying any applicable article publishing charge ("APC") for Open Access articles. Where the Submitting Author wishes to make the Work available on an Open Access basis (and intends to pay the relevant APC), the terms of reuse of such Open Access shall be governed by a Creative Commons licence – details of these licences and which Creative Commons licence will apply to this Work are set out in our licence referred to above.

Other than as permitted in any relevant BMJ Author's Self Archiving Policies, I confirm this Work has not been accepted for publication elsewhere, is not being considered for publication elsewhere and does not duplicate material already published. I confirm all authors consent to publication of this Work and authorise the granting of this licence.

For peer review only

Title Page

Title: Relationships among thriving at work, organizational commitment and job satisfaction among Chinese frontline primary public health workers during COVID-19 pandemic: a structural equation model analysis

Mo Yi[1], Di Jiang[2], Jingjing Wang[1], Zeyi Zhang[1], Yuanmin Jia[1], Baosheng Zhao[1], Lei Guo[3,4], Ou Chen[1]*

Mo Yi and Di Jiang are co-first authors of the article

[1] School of Nursing and Rehabilitation, Cheeloo College of Medicine, Shandong University, Jinan, 250012, China.

[2] Department of Nursing, Peking Union Medical College Hospital, Beijing, 100730, China.

[3] Centre for Health Management and Policy Research, Shandong University, Jinan, 250012, China.

[4]School of Public Health, Cheeloo College of Medicine, Shandong University, Jinan, 250012, China

***Corresponding author:** Ou Chen, School of Nursing and Rehabilitation, Cheeloo College of Medicine, Shandong University, No.44 Wenhua West Road, Lixia District, Jinan City, Shandong Province, 250012, China.

E-mail: chenou@sdu.edu.cn, Tel: (+0086) 053188382000

Key words: Public health workers, Thriving at work, Organizational commitment, Job satisfaction, Structural equation model;

Word count

4315(excluding title page, references, figures and tables)

Acknowledgments

This study was supported by The Natural Science Foundation of China (82172543); The

For peer review only

Natural Science Foundation of Shandong Province (ZR2020MH006); The Key Research and Development Program of Shandong Province (2019GSF108198).

Relationship among thriving at work, organizational commitment and job satisfaction among Chinese frontline primary public health workers during COVID-19 pandemic: a structural equation model analysis

Abstract

Objectives: This study aims to explore the mediating effect and influence mechanism of organizational commitment on the association among thriving at work and job satisfaction among frontline primary public health workers (PHWs) in China during the COVID-19 pandemic.

Design: This study is a cross-sectional written survey.

Setting: We included 20 primary care units in northern provinces of China.

Participants: A total of 601 PHWs who worked in primary organizations and against COVID-19 on the frontline were included.

Methods: We collected the data from the participants' written questionnaire (Minnesota Satisfaction Questionnaire, thriving at work scale, and organizational commitment scale), and programmed AMOS 26.0 to develop a structural equation model (SEM) based on the relationships among the three variables.

Results: The thriving at work scores of the primary PHWs were (M=3.17, SD=0.65), and job satisfaction was (M=3.05, SD=0.69); the scores of their thriving at work, organizational commitment and job satisfaction were all significantly correlated (P<0.01); and the SEM indicated that organizational commitment had a significant partial mediating effect between thriving at work and job satisfaction. The overall effect value was 0.867, and the mediated effect value was 0.422, accounting for 48.7% of the total effect size.

Conclusion: The thriving at work and job satisfaction scores of primary PHWs in China are moderate, and thriving at work not only affects job satisfaction directly, but also indirectly

For peer review only

through organizational commitment. This study suggests that health policy-makers should promote job satisfaction among PHWs through relative inventions aiming to improve their thriving at work and organizational commitment.

Strengths and limitations of this study

- The structural equation model was used to analyse the associations among thriving at work, organizational commitment, and job satisfaction.
- The covariates, including participants' gender, age, years of working, and professional level, were adjusted to ensure the accuracy of the statistical analysis.
- Only primary PHWs in the eastern region of China were included in the study.
- The cross-sectional nature of this study cannot-be used to deduce the causal relationships among the three variables.
- This study focused more specifically on the frontline pandemic-fighting population of primary public health workers.

1.Introduction

In early 2020, an epidemic called COVID-19 suddenly swept the world, ultimately causing millions of infections and deaths worldwide. The WHO identified the outbreak as a Public Health Emergency of International Concern (PHEIC), which poses a huge threat to people's health and the global economy[1, 2]. As lower-level medical organizations, China's primary health care units played an important role in the battle against COVID-19 on the frontline of defence[3].

Public health is a significant part of China's health care service system and an indispensable force in improving the health of community residents. Primary PHWs are responsible for the detection, prevention and control of various infectious diseases, occupational health examinations, planned immunizations and epidemiological surveys[4, 5]. In China, public health workers (PHWs) are a separate group of health care providers, different from other professional groups, such as physicians, pharmacists and nurses. They are mainly responsible for epidemiological investigations, quarantine management of patients with infectious diseases, environmental disinfections, and specimen collections in the community

For peer review only

or other primary organizations. As the essential gatekeepers in epidemic control, since the outbreak, primary PHWs have not only completed heavy workloads, but also risked being infected while working on the frontline[6, 7]. They are in a state of chronic psychological stress and gradually experience emotional exhaustion from their profession. In addition, their personal physical, energetic, and motivational strengths are in a state of increasing overload, which has negative effects on their job satisfaction and thriving at work[8].

Job satisfaction is the subjective psychological state of employees' physiological and psychological perceptions of the work environment and the work itself[9]. Vladica's empirical study subdivided job satisfaction into internal and external satisfaction[10]. Currently, job satisfaction is used as the best predictor to measure employees' self-fulfilment of their professional values[11,12]. Unlike the development of large hospitals, primary care units in China are faced with multiple challenges, such as smaller platforms with limited opportunities, employees’ lower salaries, and weaker medical service delivery capacities[13]. PHWs have not received much attention for a long time, compared with physicians and nurses. This is why our study focuses on the current status of job satisfaction and, thriving at work of primary PHWs with their occupation and its environment.

Thriving at work is a positive psychological state characterized by a joint sense of vitality and learning, that ultimately contributed to the flourishing development of the organization according to Spreitzer[14]. A socially embedded model of thriving at work suggests that vitality represents the hedonic component of well-being, whereas learning, is a means of realizing one’s potential, which indicates job satisfaction is an external display of thriving at work[15]. Thriving at work is a crucial factor influencing health care providers’ personal career development and planning, accordingly contributing to their professional fulfillment and maintaining health[16]. Zhao’s study pointed out that healthcare providers in China who lacked thriving at work often experienced low job satisfaction, which affected their work enthusiasm and stability[17]. This indicates a highly positive correlation between job satisfaction and thriving at work, both of which are directly related to the level of quality of health care services[5, 18].

Organizational commitment usually refers to the relative strength of an individual's identification with and involvement in a particular organization[19]. Meyer and Allen argued

For peer review only

that organizational commitment is a psychological phenomenon that encourages employees to stay with the organization and participate wholeheartedly in its work as their "side-bet"; they regard it as consisting of three components: affective, continuance, and normative commitment[20]. The level of organizational commitment increases from intrinsic value, which is another key expression of thriving at work. A recent study by Kleine confirmed a positive relationship between thriving at work and organizational commitment (RC=0.63)[21]. A study that concentrated on nurses noted that organizational commitment was a significant positive predictor of their job satisfaction[22]. In addition, researchers confirm that each component of organizational commitment is a critical determinant that affects an individual's job satisfaction and life satisfaction[23, 24].

In the field of occupational research and medical practice, the job demands-resources (JD-R) model proposed by Bakker recently attracted attention with regard to creating organizational culture, improving employee job satisfaction, and reducing turnover during the pandemic[25]. Job demands usually refer to the extent to which the duties of the job require employees to exert their own somatic, social or psychological (cognitive and emotional) efforts, such as workload, emotional demands and hostile work environment. Job resources, on the other hand, consist of a set of components that are beneficial or supportive to the employee, mainly in terms of intrinsic motivation, social praise or organizational belonging[26]. The JD-R model has been tested and validated in a large sample of different countries and industries[27-29]. We consider the increased workload and physical and mental exhaustion of primary PHWs during an epidemic as high job demands, and employees' organizational commitment and perceived sense of thriving as job resources. Job demands and job resources interact mutually and are reflected in the job satisfaction which is the main outcome. Therefore, the model needs to be used to further explore what factors of job demand (resources) are associated with job satisfaction.

It is known that job satisfaction, thriving at work and organizational commitment are the key factors that affect employee job stability and that the individual has a stable job for a certain period of time and does not have the idea of leaving easily[30]. However, the inherent influence mechanism among the three variables of PHWs is still unclear. Most existing studies have explored the job satisfaction of physicians or nurses in large hospitals, with less

For peer review only

attention given to the public health personnel group. Therefore, this study is equipped with some scientific research value for human resources in the field of PHWs. Therefore, the first aim of this study was to assess the current status of thriving at work and job satisfaction among primary PHWs during the COVID-19 pandemic. The second aim was to construct a structural equation model(SEM) for these three variables among primary PHWs, empirically analyse their quantitative relationships, and provide suggestions on the relevant policies and health care practices, because it is crucial to stabilize the workforce of primary PHWs by improving their job satisfaction. Meanwhile, we hypothesized that (1) thriving at work and organizational commitment are protective factors for job satisfaction among primary PHWs, and (2) thriving at work indirectly and directly affects job satisfaction through organizational commitment.

2. Methods

2.1 Study participants

This study was a cross-sectional study. A convenience sampling method was used to determine the 20 primary health care units (community/township health service centres, rural clinics) in northern provinces of China with whom we had a partnership, and the random number table was used to determine the targeting of 650 primary PHWs from May to October 2020.

The inclusion criteria for participants were as follows: (1) years of work in primary care units ≥ 1 year; (2) having participated in frontline work against the COVID-19 epidemic for more than 1 month; and (3) professional qualification as a licenced registered PHW. The exclusion criteria were as follows: (1) interns and trainees; (2) those whose work sites were nonprimary organizations; and (3) physicians or nurses. A total of 650 questionnaires were distributed in this survey, and 601 valid questionnaires were finally returned, for a valid return rate of 92.46%.

2.2 Measures

2.2.1 Sociodemographic information questionnaire

After referring to previous literature and expert consultation, we identified the following

For peer review only

demographic information as the study variables: gender, age, education background, type of working unit (community/township healthcare service centres, rural clinics), years of working and professional level of the participants.

2.2.2 Minnesota Satisfaction Questionnaire

The Minnesota Satisfaction Questionnaire (MSQ), a short-form scale developed by Weiss, Dawis, England and Lofquist to measure employee job satisfaction, contains two dimensions: internal and external satisfaction[31]. The MSQ has 20 items and is scored on a 5-point Likert scale, with "very dissatisfied", "dissatisfied", "fair", "satisfied", "very satisfied" and "satisfied". The higher the score is, the higher the satisfaction level of primary PHWs with their work. Previous studies focused on health care providers have shown that the Cronbach's alpha value of this scale is 0.87-0.92[32, 33].

2.2.3 Organizational Commitment Questionnaire

The Organizational Commitment Questionnaire (QCQ), developed by Allen and Meyer[34], was used in this study to measure the organizational commitment of primary PHWs. It contains three dimensions: affective commitment, normative commitment, and sustaining commitment, and each dimension includes six items. All items are measured on a Likert 5-point scale from 1 (strongly disagree) to 5 (strongly agree). The Cronbach's alpha values for the dimensions are 0.928, 0.802, and 0.859[35], respectively. The total scale score was the sum of the items and ranged from 18 to 90, with higher scores representing higher levels of employee organizational commitment[36].

2.2.4 Thriving at Work Scale

The thriving at work scale developed by Porath[37] was used; it has 10 items and measures two dimensions of thriving at work, vitality and learning, with each containing 5 items. This is a Likert 5-point scale from "1 to 5" representing "very unlikely ~ very likely", respectively. The higher the score is, the higher the sense of thriving at work of the participants. The Chinese version of the questionnaire has been shown to have good reliability and validity in the Chinese teacher and nurse populations[38, 39].

For peer review only

2.3 Data collection

This study was an anonymous and written survey, and all the data obtained were promised confidentiality. Rigorous training and assessment of the investigators were conducted before the survey began, and the participants all signed an informed consent prior to the study. The paper questionnaires were distributed together by the investigators in cooperation with the leaders of the units or organizations, and the participants received instructions. The questionnaires were carefully checked by the investigators after the participants had completed them and were collected in time. This study was approved by the Ethics Committee of Shandong University on 15th July 2020 in China, with the approval No. 2020-R01-23.

2.4 Statistical analysis

SPSS version 21.0 was used to analyse the data descriptively. A t test and one-way ANOVA were used to compare the differences among variables on the sociodemographic characteristics of the study population. Pearson correlation analysis was used to clarify the correlations among organizational commitment, job satisfaction and thriving at work among primary PHWs, with a p value smaller than 0.05 considered a statistically significant difference. Based on the correlation analysis, AMOS 26.0 was used to develop an SEM to analyse the inherent relationships among the three variables. The path coefficients between the latent variables were tested using path analysis, and the parameter estimation was performed using the great likelihood method, assuming that the significance of the mediating effect of organizational commitment in the model was calculated using bootstrap analysis with 2,000 samples with the test level set at $\alpha=0.05$, and if the 95% confidence interval of the standardized path coefficients did not contain 0, the mediating effect was significant. The SEM can be considered well-fitted when all path coefficients satisfy the criteria of Hu and Bentler's study[40].

3. Results

3.1 General information

For peer review only

A total of 650 primary PHWs who had participated on the frontline of epidemic fighting were surveyed in 2020. The average age of the participants was 36.61±8.95 years old, with the majority being female (82.9%). The education background of all participants was mainly college or above (94.0%). The average working years was 14.31±6.21 years, and their professional levels, which is a comprehensive reflection of their work experience and work ability, were mainly junior (72.3%). For the three main scales involved in this study, we performed the relevant Cronbach coefficient tests, and the results were as follows: (1) the Cronbach's alpha value of the Minnesota Satisfaction Questionnaire was 0.919; (2) the Cronbach's alpha value of the Organizational Commitment Questionnaire in this study was 0.917; (3) the overall Cronbach's alpha value of the thriving at work scale was 0.89, and the Cronbach′s alphas for the vitality and learning dimensions were 0.87 and 0.89, respectively.

3.2 Analysis of common method bias test

In this study, a common method bias test was conducted using Harman's one-way test and exploratory factor analysis was performed without rotation. The results showed that there were 14 factors with characteristic roots greater than 1. The variance rate explained by the first factor of job satisfaction was 36.73%, which was less than the critical value of 40%, indicating that there was no serious problem of common method bias in this study[41].

3.3 Demographic differences in thriving at work and job satisfaction scores

The results showed that for primary PHWs, there were significant differences in thriving at work on two demographic variables: education and years of working. The PHWs who had obtained a lower education level had higher thriving at work; additionally, the lowest level of thriving at work was observed in the group with more than 20 years of working.

There were significant differences in job satisfaction among primary PHWs on three demographic variables: gender, education and years of working. Female PHWs had higher scores than males in job satisfaction; PHWs with more years of working had a higher level of job satisfaction; and PHWs with a better education background had lower job satisfaction, as detailed in **Table 1**.

Table 1 Demographic differences in thriving at work and job satisfaction

For peer review only

among primary PHWs

Variables	Scores of TW		Scores of JS	
	scores	*t/F* value	scores	*t/F* value
**Gender**		0.762		1.825**
Male	3.13±0.70		3.91±0.80	
Female	3.18±0.64		4.07±0.67	
**Age**		0.126		0.066
<30 years old	3.16±0.74		3.04±0.74	
30-45 years old	3.19±0.59		3.06±0.67	
>45 years old	3.16±0.63		3.05±0.68	
**Years of working**		0.267*		1.873*
<10 years	3.15±0.69		3.05±0.72	
10-20 years	3.20±0.61		3.03±0.68	
>20 years	3.17±0.67		3.06±0.66	
**Educational level**		0.396*		0.785**
Diploma	3.16±0.54		3.08±0.57	
Bachelor's degree	3.12±0.65		3.02±0.68	
Master's degree	3.05±0.61		3.96±0.46	
**Professional title**		0.311		0.655
Junior staff	3.05±0.72		3.05±0.72	
Middle staff	3.03±0.68		3.03±0.68	
Senior staff	3.04±0.66		3.06±0.66	

Abbreviations: TW, thriving at work; OC, organizational commitment; JS, job satisfaction.

Note: **P*< 0.05, ***P*< 0.01. Professional title is a comprehensive reflection of the registered public health workers' work ability and work position in the related field.

3.4 Correlations among organizational commitment, job satisfaction and thriving at work among primary PHWs

The results showed that the mean score of thriving at work of the 601 responding primary PHWs was 3.17±0.65. The mean score of their organizational commitment was 2.93±0.73. The mean score of their job satisfaction was 3.05±0.69. Pearson's correlation analysis revealed that thriving at work was positively correlated with organizational

For peer review only

commitment (r=0.715, P<0.001) and highly positively correlated with job satisfaction (r=0.804, P<0.001); furthermore, there was a highly positive correlation between organizational commitment and job satisfaction (r=0.848, P<0.001) (see **Table 2)**.

Table 2 Correlations among thriving at work, organizational commitment and job satisfaction among primary PHWs

Variables	Mean ± SD	TW	OC	JS
TW	3.17±0.65	1	—	—
OC	2.93±0.73	0.715**	1	—
JS	3.05±0.69	0.804**	0.848**	1

Abbreviations: TW, thriving at work, OC, organizational commitment, JS, job satisfaction

Note: *P< 0.05, **P< 0.01.

3.5 SEM of organizational commitment, job satisfaction, and thriving at work among primary PHWs

To investigate the influence mechanism among the variables in depth, we controlled for the gender, age, education background, and years of working of the participants. An SEM was constructed with thriving at work as the independent variable, organizational commitment as the mediating variable, and job satisfaction as the dependent variable. AMOS 26.0 software was used to conduct the validation analysis of the hypothesized model, and the results showed that each path coefficient was statistically significant (p<0.001), as shown in **Figure 1**. The model fit statistics were: chi-squared degrees of freedom (χ^2/df) =3.416, GFI=0.982, AGFI=0.953, NFI=0.993, CFI=0.995, IFI=0.995, and RMSEA=0.063 (95% confidence interval 0.042, 0.086). All the above fit indices are within the acceptable range and the model fits well, as shown in **Table 3**.

Abbreviations: CC, continuance commitment; NC, normative commitment; AC, affective commitment; IS, internal satisfaction; ES, external satisfaction.

For peer review only

Figure 1 Structural equation model of primary PHWs' thriving at work, organizational commitment, and job satisfaction

Table 3 Index evaluation system and fitting results of structural equation model adaptability

Fit Index	χ^2/df	GFI	AGFI	RMSEA	IFI	CFI	NFI
Theoretical Model	＜3	＞0.9	＞0.9	＜0.08	＞0.9	＞0.9	＞0.9
Regression Criterion	3.416	0.982	0.953	0.063	0.995	0.995	0.993

Abbreviations: *df*, degree of freedom; GFI, goodness-of-fit index; AGFI, adjusted goodness-of-fit index; RMSEA, root-mean-square error of approximation; IFI: incremental fit index; CFI, comparative fit index; NFI, normed fit index.

For peer review only

The bootstrap method was used to test the significance of the mediating role of organizational commitment between thriving at work and job satisfaction. Repeated samples were taken 2,000 times, and 95% confidence intervals were calculated. The results showed that the standardized effect value of the mediating effect of organizational commitment between thriving at work and job satisfaction was 0.422, 95% *CI* (0.276, 0.561), and the standardized effect value of the total effect was 0.867, 95% *CI* (0.781, 0.920), neither of which contained 0, indicating that the differences in all effect paths were statistically significant. The calculated mediated effect size was 48.7% of the total effect size (that is, the indirect effect value as a percentage of the total effect value), and the specific path coefficients of the model are shown in **Table 4**.

Table 4 Pathway coefficients of structural equation model (n=601)

Pathways	Std. estimate	95% *CI*	SE	*P* value
**Total effect**				
	0.867	(0.781,0.920)		0.002**
**Direct effect**				
TW→JS	0.445	(0.269,0.640)	0.098	0.001**
TW→OC	0.765	(0.662,0.844)	0.046	＜0.01**

OC→JS	0.552	(0.364,0.726)	0.096	0.001**
**Indirect effect**				
TW→OC→JS	0.422	(0.276,0.561)	0.070	0.001**

Abbreviations: TW, thriving at work; OC, organizational commitment; JS, job satisfaction; Std. Estimate, standardized estimate; CI, confidence interval; SE, standard error.

Note: *P< 0.05, **P< 0.01.

4. Discussion

Based on the JD-R model, this study found that during the COVID-19 pandemic, primary PHWs in China had a moderate level of job satisfaction. Primary PHWs with adequate thriving at work are more likely to exhibit higher job satisfaction. In addition, thriving at work also can indirectly influence PHWs' job satisfaction through organizational commitment.

The current status of thriving at work and job satisfaction among primary PHWs

Job satisfaction is an indispensable indicator of the quality of work performed by primary PHWs and has an important impact on their physical and mental health development[42]. However, during the pandemic in China, when small-scale outbreaks occurred in some areas, primary PHWs were on the frontline of the fight against the epidemic and were required to respond quickly to their areas of responsibility[43]. Consequently, they were exposed to greater workload, physical injuries and mental stress caused by higher work demands, with insufficient work resources, which seriously deteriorated their job satisfaction[44]. The results showed that the thriving at work score of primary PHWs was 3.17±0.65. In addition, the score of job satisfaction was 3.05±0.69, which was lower than the scores of PHWs in Nigeria and Iran[45, 46]. Meanwhile, the results of the ANOVA showed that there were statistically significant differences in the job satisfaction scores of primary PHWs in terms of gender, years of working, and educational background. That is, primary PHWs who are male, have higher education, or have fewer years of working experience were at a relatively lower level of job satisfaction. The potential explanations could be the fact that grassroots positions offer fewer promotion and self-development opportunities, inadequate

For peer review only

salaries and insufficient supportive measures, resulting in primary PHWs with master's degrees or higher being unable to give full play to their knowledge in health care practice, thus limiting their long-term career development and causing lower job satisfaction[47]. In addition, when employees have fewer years of working experience, their coping ability is still in the ascending stage, it is not possible to be very proficient in work operations and have limited access to work resources, especially in the heavy epidemic period, which to a certain extent reduces their job satisfaction[48].

Correlations and SEM among organizational commitment, thriving at work, and job satisfaction among primary PHWs

Previous studies have shown a positive relationship between organizational commitment and job satisfaction among various industries, while such a relationship has not been confirmed among primary PHWs[49]. First, the results of Pearson analysis showed that thriving at work has a significantly positive correlation with job satisfaction (β=0.804, P<0.01). The integrated model of personal growth of 'thriving at work' suggests that a key output of thriving at work is the indicator of job satisfaction[50]. A meta-analysis that included 21,739 subjects found that teachers with a high level of thriving at work exhibited greater job satisfaction[51]. In addition, thriving at work is one of the significant factors of work resources for health care occupations and health-related organizations. An individual's thriving focuses on two domains, vitality and learning, in daily work[52]. Primary PHWs can enhance learning through various types of training, to create a positive work atmosphere that is highly beneficial to both individuals and health care organizations. Moreover, vitality can help individuals adapt creatively to the difficulties of some situations, especially during the risky and challenging COVID-19 pandemic[53]. Second, organizational commitment maintained a highly positive relationship with job satisfaction(β =0.848, P<0.01). PHWs with high organizational commitment have a deep attachment to their health care units and a strong sense of responsibility and mission for their jobs. The correlations among the three components of organizational commitment and job satisfaction can be reflected as follows: if the primary PHWs exhibit higher affective commitment, they may have greater identification

For peer review only

with the organization's goals, values and culture, and higher satisfaction with their job[54]. Additionally, continuance commitment facilitates primary PHWs' loyalty to the organization, which in turn enhances their satisfaction. Normative commitment ensures that the primary PHWs believe that they have a great responsibility to the organization, and the greater the psychological satisfaction that active work can bring to the individual, the higher their job satisfaction[55]. Finally, there is a positive relationship between thriving and organizational commitment, and thriving on the content, atmosphere, and nature of their work promotes employees' attachment to the organization, they are more likely to derive higher satisfaction from their work[56].

The results of the SEM in this study also confirmed that organizational commitment partially mediated the relationship between thriving at work and job satisfaction, with a mediating effect size of -0.35, accounting for 57.38% of the total effect, indicating that organizational commitment serves as a significant factor affecting primary PHWs' level of job satisfaction. This is consistent with the result of Malgorzata's study, but his study participants were cross-cultural workers[57]. Therefore, the new relationships among thriving at work, organizational commitment and job satisfaction confirmed in this study offer the first validation in a group of Chinese health care providers. Furthermore, Bakker's study supports that organizational commitment is the result of a combination of many different job demands and job resources. Positive organizational commitment can have an impact on job satisfaction[58]. According to psychological contract theory, the cognitive structure of individuals determines how public health workers view their relationship with the organization. This is considered to be their belief system, the obligations that exist between them and their work health care organization[59]. Therefore, the mechanisms underlying the relationship indicate that thriving at work is a state of need and motivation that is critical to the internal psyche of the individual and that is easily activated and motivated by situational factors. A theoretical model of thriving at work developed by Spreitzer and colleagues[60] suggests that when employees feel energized and full of learning in the workplace, they are energized and willing to invest more time, energy, and effort in their work, resulting in a high level of thriving at work. This will further stimulate their identification with their organizations (i.e., affective commitment), which will lead to a higher level of centripetal

For peer review only

force towards their careers and the groups they work with (i.e., continuance commitment), and a greater willingness to engage in organizational activities and construction out of their own strong sense of responsibility and mission (i.e., normative commitment)[61]. Additionally, employees' commitment to the organization inevitably affects employees' attitudes or feelings toward work, which in turn affects their job satisfaction. Positive work can bring psychological satisfaction to the individual, so the higher the employee's job satisfaction is, the higher the satisfaction with the job content, the nature of the job, and the atmosphere of the job.

This study explored the intrinsic association between thriving at work and job satisfaction by constructing an SEM with organizational commitment as a mediating variable. Thriving has been proven to promote job sustainability among primary PHWs and can have a direct impact on their job satisfaction, as well as an indirect impact on their job satisfaction by increasing the level of organizational commitment. This study provides a theoretical basis for better stabilizing the talent pool of primary PHWs in China, improving the service quality of primary public health and medical care in China, and optimizing the allocation of primary medical resources.

Implications on policies or practices

The suggestions on the policies and practices can be made from the perspective of health policy-makers, as well as from that of the management of leaders. First, the health policymakers are advised to refer to the way of magnet hospitals, although magnet hospitals were originally applied to nursing initiatives to ameliorate the environment in which nurses practise. Specifically, policy-makers need to focus on providing a platform for staff career development, as well as providing supportive continuing education. Establishing a scientific and reasonable salary system and creating a positive organizational culture are conducive to mobilizing and motivating primary PHWs, thus improving their job satisfaction. Second, for the leadership of employers, leaders should fully explore the potential advantages of primary PHWs in thriving at work, establish a relationship of mutual trust with their subordinates, respond positively to their opinions and suggestions, and cultivate their sense of organizational identity. Consequently, primary PHWs are more willing to integrate the

For peer review only

organization's development goals into their personal values and ideals, thus generating a higher level of organizational commitment, which is an intrinsic motivation for their proactive efforts.

Study limitations

There are some limitations that cannot be ignored in this study. First, participants may have had recall bias when completing the scales. Second, we did not use a weighting method by the size of the provinces in the selection of primary health care units, which could be a potential source of sample selection bias. Third, physicians and nurses were the main participants in the fight against COVID-19 in other countries. This study has some implications for other countries, but the limitation in the generalization of the results needs to be noted because of the differences in the target groups. Finally, the relationships among variables found in this cross-sectional study do not necessarily reflect causality. Future longitudinal studies with larger samples and incorporating more variables are needed to validate the results of this study.

5. Conclusion

This study reveals that Chinese primary PHWs face high job demands and limited job resources, leading to their poor thriving and job satisfaction. Meanwhile SEM results found that thriving at work has a direct contribution to job satisfaction, while organizational commitment as a mediating variable also can increase job satisfaction. Therefore, in the context of the COVID-19 pandemic, health policy-makers should pay high attention to this group of primary PHWs and enhance their thriving and organizational commitment by regulating their job demands and providing sufficient job resources to effectively activate their job satisfaction and fulfilment, which is essential to maintain the job stability of primary PHWs.

Author Contributions

Conceived and designed the research: Mo Yi, Di Jiang, Ou Chen.

For peer review only

Performed the research: Yuanmin Jia, Jingjing Wang, Lei Guo, Zeyi Zhang.

Analyzed the data: Mo Yi, Yuanmin Jia, Mo Yi, Di Jiang

Contributed reagents/materials/analysis tools: Yuanmin Jia, Baosheng Zhao.

Wrote the manuscript: Mo Yi.

Mo Yi and Di Jiang are co-first authors of the article

Acknowledgments

We are particularly grateful to the coordinators who assisted us, especially all the primary PHWs who participated in this study and supported the study during the busy period of pandemic.

Funding

This study was supported by The Natural Science Foundation of China (82172543); The Natural Science Foundation of Shandong Province (ZR2020MH006); The Key Research and Development Program of Shandong Province (2019GSF108198).

Disclosure

The authors report no conflicts of interest in this work.

Data availability statement

The data or models generated or used in the study are available from the corresponding author by reasonable request.

Patient and Public Involvement

Patients and/or the public were not involved in the design, or conduct, or reporting or dissemination plans of this research.

Ethics statement

The study was approved by the ethic board of School of Nursing and Rehabilitation, Shandong University, China. All participants provided written informed consent.

For peer review only

References:

1. Lancet T. COVID-19: too little, too late? *Lancet (London, England).* 2020(No.10226):755.

2. Liu C, Zhao Y, Okwan-Duodu D, Basho R, Cui X. COVID-19 in cancer patients: risk, clinical features, and management. *Cancer Biol Med.* 2020(No.3):519-527.

3. Wang H, Tang C, Zhao S, Meng Q, Liu X. Job Satisfaction among Health-Care Staff in Township Health Centers in Rural China: Results from a Latent Class Analysis. *Int J Environ Res Public Health.* 2017;14(10).

4. Shi LSL, Song KSK, Rane SRS, Sun XSX, Li HLH, Meng QMQ. Factors associated with job satisfaction by Chinese primary care providers. *Prim Health Care Res.* 2014(No.1):46-57.

5. Zhou J, Lee DM, Yiqi J, Xuan J. Thriving at work: A mentoring-moderated process linking task identity and autonomy to job satisfaction. *J Vocat Behav.* 2020;118(C).

6. Li J, Xu J, Zhou H, et al. Working conditions and health status of 6,317 front line public health workers across five provinces in China during the COVID-19 epidemic: a cross-sectional study. *Bmc Public Health.* 2021;21(1):106.

7. Li J, Xu J, Zhou H, et al. Working conditions and health status of 6,317 front line public health workers across five provinces in China during the COVID-19 epidemic: a cross-sectional study. *Bmc Public Health.* 2021;21(1).

8. Jing Zhang YWJX. Prevalence of mental health problems and associated factors among front-line public health workers during the COVID-19 pandemic in China: an effort-reward imbalance model-informed study. *BMC psychology.* 2021(No.1):55.

9. Kuo HT, Lin KC, Li IC. The mediating effects of job satisfaction on turnover intention for long-term care nurses in Taiwan. *J Nurs Manag.* 2014;22(2):225-233.

10. Veličković VMA, Višnjić AAB, Jović SA, et al. Organizational commitment and job satisfaction among nurses in Serbia: A factor analysis(Article). *Nurs Outlook.* 2014(No.6):415-427.

11. Eacute P, Maximilian, Haefner N, Golden T. Clarifying CSE on Job Performance: The

For peer review only

Roles of Innovative Work Behavior & Transformational Leaders. *Academy of Management Annual Meeting Proceedings.* 2018(No.1):1.

12. Zhou J, Lee DM, Yiqi J, Xuan J. Thriving at work: A mentoring-moderated process linking task identity and autonomy to job satisfaction. *J Vocat Behav.* 2020;118(C).

13. Wang LWL, Wang ZWZ, Ma QMQ, Fang GFG, Yang JYJ. The development and reform of public health in China from 1949 to 2019. *Globalization & Health.* 2019(No.1):45.

14. Spreitzer GCFP, Sutcliffe KCFP, Dutton JCFP, Sonenshein SCFP, Grant AMCF. A Socially Embedded Model of Thriving at Work. *Organ Sci.* 2005(NO.5):537-549.

15. Abid G, Khan B, Hong MC. Thriving at Work: How Fairness Perception Matters for Employee's Thriving and Job Satisfaction. *Academy of Management Annual Meeting Proceedings.* 2016(No.1):1.

16. Abid GGAH, Zahra IIZY, Ahmed ADNE. Mediated Mechanism of Thriving at Work between Perceived Organization Support, Innovative Work Behavior and Turnover Intention. *Pakistan Journal of Commerce & Social Sciences.* 2015(No.3):982-998.

17. Zhao SH, Shi Y, Sun ZN, et al. Impact of workplace violence against nurses' thriving at work, job satisfaction and turnover intention: A cross-sectional study. *J Clin Nurs.* 2018;27(13-14):2620-2632.

18. Zhang S, Shi Y, Liu B, et al. Job demands and resources and their relationship with satisfaction and thriving at work in a sample of Chinese doctors: a cross-sectional study. *Bmj Open.* 2021;11(11).

19. Güleryüz GHUD, Güney SHUD, Aydın EMHU, Aşan ÖHUD. The mediating effect of job satisfaction between emotional intelligence and organisational commitment of nurses: A questionnaire survey. *Int J Nurs Stud.* 2008(No.11):1625-1635.

20. Meyer JP, Allen NJ. A three-component conceptualization of organizational commitment. *Hum Resour Manage R.* 1991(NO.1):61-89.

21. Kleine AKAA, Rudolph CWA, Zacher HAHZ. Thriving at work: A meta - analysis. *J Organ Behav.* 2019(No.9-10):973-999.

22. Aharon AA, Madjar B, Kagan I. Organizational commitment and quality of life at work among public health nurses in Israel. *Public Health Nurs.* 2019(No.4):534-540.

23. Culibrk JCJ, Delic MDM, Mitrovic SMS, Culibrk DCD. Job Satisfaction, Organizational

Commitment and Job Involvement: The Mediating Role of Job Involvement. *Front Psychol.* 2018:132.

24. Eric G Lambert RTSE. The Association of Organizational Justice with Job Satisfaction and Organizational Commitment among Nigerian Correctional Staff. *Int J Offender Ther.* 2021(No.2-3):180-204.

25. Demerouti E, Bakker AB. The Job Demands-Resources model: challenges for future research. *SA Journal of Industrial Psychology.* 2011;37(2):1-9.

26. Zis P, Anagnostopoulos F, Sykioti P. Burnout in medical residents: a study based on the job demands-resources model. *TheScientificWorldJournal.* 2014;2014:673279.

27. Bakker AB, Demerouti E, Taris TW, Schaufeli WB, Schreurs PJG. A multigroup analysis of the job demands-resources model in four home care organizations. *Int J Stress Manage.* 2003;10(1).

28. Zeng W, Ma S, Callan VJ, Wu L. Exploring the doctor-patient relationship as a challenge job demand: application of the job demands-resources model in a Chinese public hospital. *Psychology, health & medicine.* 2021.

29. Sabine K, Andreas R. A job demands-resources perspective on salespersons' market intelligence activities in new product development. *Journal of Personal Selling & Sales Management.* 2016;36(1).

30. Howes LMA, Goodman-Delahunty JB. Predicting Career Stability and Mobility: Embeddedness and Boundarylessness(Article). *J Career Dev.* 2015(No.3):244-259.

31. Weiss DJ, Dawis RVE, G. W. And Lofquist LH. *Manual for the Minnesota Satisfaction Questionnaire*. Vol. 22 ed. Industrial Relations Center: Minnesota Studies in Vocational Rehabilitation, Minneapolis: University of Minnesota; 1967.

32. Li N, Zhang L, Xiao G, Chen J, Lu Q. The relationship between workplace violence, job satisfaction and turnover intention in emergency nurses. *Int Emerg Nurs.* 2019:50-55.

33. Zhang X, Bian L, Bai X, et al. The influence of job satisfaction, resilience and work engagement on turnover intention among village doctors in China: a cross-sectional study. *Bmc Health Serv Res.* 2020;20(1):283.

34. Allen NJUW, Meyer JP. Affective, continuance, and normative commitment to the organization: An examination of construct validity. *J Vocat Behav.* 1996(No.3):252-276.

For peer review only

35. NGUYEN HM, NGO TT. Psychological Capital, Organizational Commitment and Job Performance: A Case in Vietnam. *The Journal of Asian Finance, Economics and Business.* 2020:269-278.

36. Lin WQA, Yuan LXB, Kuang SYB, et al. Work engagement as a mediator between organizational commitment and job satisfaction among community health-care workers in China: a cross-sectional study. *Psychology, Health and Medicine.* 2020(No.6):666-674.

37. Porath CA, Spreitzer GASU, Gibson CA, Garnett FGA. Thriving at work: Toward its measurement, construct validation, and theoretical refinement. *J Organ Behav.* 2012(No.2):250-275.

38. Yi Yang XL. The Impact of Challenge and Hindrance Stressors on Thriving at Work Double Mediation Based on Affect and Motivation. *Front Psychol.* 2021:613871.

39. X Zhu WKSS. A causal model of thriving at work in Chinese nurses. *Int Nurs Rev.* 2021:1.

40. Hu L, Bentler PM. Fit indices in covariance structure modeling: Sensitivity to underparameterized model misspecification. *Psychol Methods.* 1998(No.4):424-453.

41. Doty DHSU, Glick WH. Common Methods Bias: Does Common Methods Variance Really Bias Results? *Organ Res Methods.* 1998(NO.4):374-406.

42. Warr P, Inceoglu I. Job Engagement, Job Satisfaction, and Contrasting Associations With Person-Job Fit.[Article]. *J Occup Health Psych.* 2012(No.2):129-138.

43. Huang J, Liu F, Teng Z, et al. Care for the Psychological Status of Frontline Medical Staff Fighting Against Coronavirus Disease 2019 (COVID-19). *Clin Infect Dis.* 2020;71(12):3268-3269.

44. She R, Wang X, Zhang Z, et al. Mental Health Help-Seeking and Associated Factors Among Public Health Workers During the COVID-19 Outbreak in China. *Front Public Health.* 2021;9:622677.

45. Adeniji AA, Osibanjo AO, Iruonagbe TC, Olawande T, Ibidunni AS, Olokundun MA. From job satisfaction to organizational commitment: The mediating influence of perceived treatment of diversity among nigeria’s public healthcare employees(Article). *Open Access Macedonian Journal of Medical Sciences.* 2019(No.12):2031-2035.

46. Sarabi RE, Javanmard R, Shahrbabaki PM. Study of burnout syndrome, job satisfaction

For peer review only

and related factors among health care workers in rural areas of Southeastern Iran. *AIMS Public Health.* 2020(No.1):158-168.

47. Li L, Hu H, Zhou H, et al. Work stress, work motivation and their effects on job satisfaction in community health workers: a cross-sectional survey in China. *Bmj Open.* 2014;4(6):e4897.

48. Lu YLY, Hu XHX, Huang XHX, et al. Job satisfaction and associated factors among healthcare staff: a cross-sectional study in Guangdong Province, China. *Bmj Open.* 2016(No.7):e11388.

49. Ghulam A, Francoise C, Saira A, Tehmina Q. Contextual Factors and Organizational Commitment: Examining the Mediating Role of Thriving at Work. *Sustainability-Basel.* 2019;11(17).

50. Spreitzer GM, Porath CL. Self-Determination as a Nutriment for Thriving: Building an Integrative Model of Human Growth at Work. *The Oxford Handbook of Work Engagement, Motivation, and Self-Determination Theory.* 2014.

51. Kleine AKAA, Rudolph CWA, Zacher HAHZ. Thriving at work: A meta ‐ analysis. *J Organ Behav.* 2019(No.9-10):973-999.

52. Danping Liu SZYW. The Antecedents of Thriving at Work: A Meta-Analytic Review. *Front Psychol.* 2021:659072.

53. Ding YDY, Qu JQJ, Yu XYX, Wang SWS. The Mediating Effects of Burnout on the Relationship between Anxiety Symptoms and Occupational Stress among Community Healthcare Workers in China: A Cross-Sectional Study. *Plos One.* 2014(No.9):e107130.

54. Yang F, Chang C. Emotional labour, job satisfaction and organizational commitment amongst clinical nurses: a questionnaire survey. *Int J Nurs Stud.* 2008;45(6).

55. Darwish AY. Job Satisfaction as a Mediator of the Relationship Between Job Stressors and Affective, Continuance, and Normative Commitment: A Path Analytical Approach. *Int J Stress Manage.* 2002;9(2).

56. Harris O, Jacobsen P, Searle-Barnes R, Wilkinson-Tough M. Surviving and thriving - a mixed-methods study of staff experiences of occupational wellbeing in a psychiatric place of safety service. *J Ment Health.* 2020.

57. Rozkwitalska M, Basinska BA. THRIVING AND JOB SATISFACTION IN

For peer review only

MULTICULTURAL ENVIRONMENTS OF MNCS. *SMART AND EFFICIENT ECONOMY: PREPARATION FOR THE FUTURE INNOVATIVE ECONOMY.* 2016:111-119.

58. Arnold BB, Marc VV, Despoina X. Beyond the Demand-Control Model: Thriving on High Job Demands and Resources. *J Pers Psychol.* 2010;9(1).

59. Ge J, He J, Liu Y, et al. Effects of effort-reward imbalance, job satisfaction, and work engagement on self-rated health among healthcare workers. *Bmc Public Health.* 2021;21(1).

60. Spreitzer G, Porath CL, Gibson CB. Toward human sustainability: How to enable more thriving at work. *Organ Dyn.* 2012;41(2SI):155-162.

61. Gielissen KA, Taylor EP, Vermette D, Doolittle B. Thriving among Primary Care Physicians: a Qualitative Study. *J Gen Intern Med.* 2021;36(12).

For peer review only

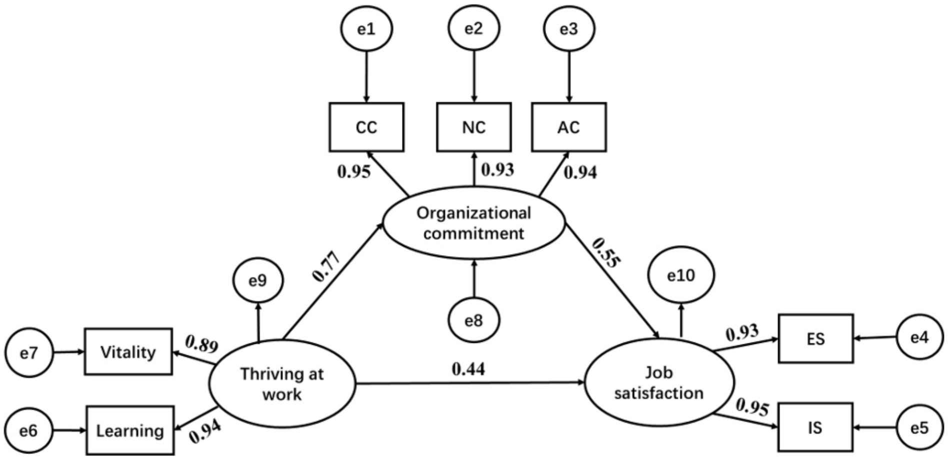

Structural equation model of primary PHWs' thriving at work, organizational commitment, job satisfaction

447x233mm (57 x 57 DPI)

STROBE Statement—Checklist of items that should be included in reports of ***cross-sectional studies***

	Item No	Recommendation	Page No
**Title and abstract**	1	(*a*) Indicate the study's design with a commonly used term in the title or the abstract	2
		(*b*) Provide in the abstract an informative and balanced summary of what was done and what was found	2
**Introduction**			
Background/rationale	2	Explain the scientific background and rationale for the investigation being reported	3
Objectives	3	State specific objectives, including any prespecified hypotheses	5
**Methods**			
Study design	4	Present key elements of study design early in the paper	5
Setting	5	Describe the setting, locations, and relevant dates, including periods of recruitment, exposure, follow-up, and data collection	5
Participants	6	(*a*) Give the eligibility criteria, and the sources and methods of selection of participants	5
Variables	7	Clearly define all outcomes, exposures, predictors, potential confounders, and effect modifiers. Give diagnostic criteria, if applicable	5,6
Data sources/ measurement	8*	For each variable of interest, give sources of data and details of methods of assessment (measurement). Describe comparability of assessment methods if there is more than one group	7
Bias	9	Describe any efforts to address potential sources of bias	7
Study size	10	Explain how the study size was arrived at	7
Quantitative variables	11	Explain how quantitative variables were handled in the analyses. If applicable, describe which groupings were chosen and why	7
Statistical methods	12	(*a*) Describe all statistical methods, including those used to control for confounding	8
		(*b*) Describe any methods used to examine subgroups and interactions	8
		(*c*) Explain how missing data were addressed	8
		(*d*) If applicable, describe analytical methods taking account of sampling strategy	8
		(*e*) Describe any sensitivity analyses	8
**Results**			
Participants	13*	(a) Report numbers of individuals at each stage of study—eg numbers potentially eligible, examined for eligibility, confirmed eligible, included in the study, completing follow-up, and analysed	9
		(b) Give reasons for non-participation at each stage	9
		(c) Consider use of a flow diagram	
Descriptive data	14*	(a) Give characteristics of study participants (eg demographic, clinical, social) and information on exposures and potential confounders	9
		(b) Indicate number of participants with missing data for each variable of interest	9
Outcome data	15*	Report numbers of outcome events or summary measures	10
Main results	16	(*a*) Give unadjusted estimates and, if applicable, confounder-adjusted estimates and their precision (eg, 95% confidence interval). Make clear which confounders were adjusted for and why they were included	10

For peer review only - http://bmjopen.bmj.com/site/about/guidelines.xhtml

		(*b*) Report category boundaries when continuous variables were categorized	11
		(*c*) If relevant, consider translating estimates of relative risk into absolute risk for a meaningful time period	11
Other analyses	17	Report other analyses done—eg analyses of subgroups and interactions, and sensitivity analyses	12,13
**Discussion**			
Key results	18	Summarise key results with reference to study objectives	13-17
Limitations	19	Discuss limitations of the study, taking into account sources of potential bias or imprecision. Discuss both direction and magnitude of any potential bias	17
Interpretation	20	Give a cautious overall interpretation of results considering objectives, limitations, multiplicity of analyses, results from similar studies, and other relevant evidence	17
Generalisability	21	Discuss the generalisability (external validity) of the study results	17
**Other information**			
Funding	22	Give the source of funding and the role of the funders for the present study and, if applicable, for the original study on which the present article is based	18

*Give information separately for exposed and unexposed groups.

Note: An Explanation and Elaboration article discusses each checklist item and gives methodological background and published examples of transparent reporting. The STROBE checklist is best used in conjunction with this article (freely available on the Web sites of PLoS Medicine at http://www.plosmedicine.org/, Annals of Internal Medicine at http://www.annals.org/, and Epidemiology at http://www.epidem.com/). Information on the STROBE Initiative is available at www.strobe-statement.org.

For peer review only
